# The Mechanism and Pathways of Formation and Modification of Salivary Metabolic Profile in Cancer

**DOI:** 10.3390/cimb48070728

**Published:** 2026-07-16

**Authors:** Elena I. Dyachenko, Lyudmila V. Bel’skaya

**Affiliations:** Biochemistry Research Laboratory, Omsk State Pedagogical University, 644099 Omsk, Russia; a.dyachenko@omgpu.ru

**Keywords:** saliva, cancer, electrolytes, amino acids, lipids, cytokines, tumor markers

## Abstract

Saliva is a promising diagnostic fluid for studying diseases, including cancer. Saliva composition can reflect both local processes occurring in the oral cavity and systemic changes associated with distant tumors. This review examines changes in salivary electrolyte, amino acid, lipid, and cytokine profiles, tumor markers, and the oral microbiome in cancer. Collectively, these aspects reflect metabolic, inflammatory, immune, secretory, and tumor-associated processes. Metabolites can enter saliva via the salivary glands, systemic circulation, gingival fluid, extracellular vesicles, and oral cells, as well as directly from the site of disease during localized pathological processes. In tumors not localized in the oral cavity, changes in saliva composition are more often associated with systemic inflammation, altered oral microbiome, metabolic reprogramming, oxidative stress, and tumor-associated exosomes. Individual metabolites have limited specificity and cannot be used as independent diagnostic indicators. A comprehensive multimarker analysis of saliva is of greatest value. This approach can facilitate early diagnosis, identify the risk of disease development and progression, monitor therapy, and understand the biological changes associated with the pathological process, including tumors.

## 1. Introduction

**Figure 1:**
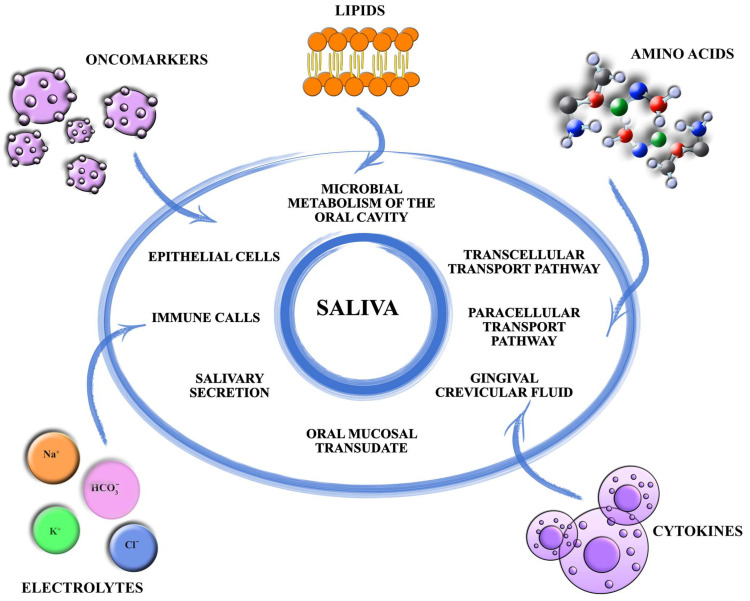
Possible sources of electrolytes, amino acids, lipids, cytokines, and tumor markers in saliva.

Saliva is increasingly attracting attention in scientific research and practical applications due to its noninvasive collection method and its ability to reflect physiological changes in the body at the local and systemic levels [1]. Compared to blood sampling, saliva collection is simple, cost-effective, and suitable for long-term patient monitoring [2]. This review examines the main pathways and mechanisms by which electrolytes, amino acids, lipids, cytokines, and tumor markers penetrate saliva, as well as the causes of changes in the oral microbiome composition and its contribution to changes in other salivary parameters (Figure 1).

Electrolytes can directly affect the function of the salivary glands, the pH of saliva, enzymatic activity, and the general condition of the oral cavity. The electrolyte profile can change indirectly in case of systemic disease or changes in the nature of salivary gland secretion [3]. Changes in the amino acid composition may indicate increased protein degradation, cell proliferation, and changes in the balance of catabolic and anabolic processes associated with cancer [4]. Lipids can reflect damage to cell membranes during an active proliferative process, inflammation, and changes in the energy balance [5,6]. Cytokines describe the immune and inflammatory reactions accompanying tumor processes [7]. Tumor markers are a more specific indicator of the disease, especially if molecules of tumor origin or mediated by the tumor process enter the saliva through the systemic circulation, extracellular vesicles, or local tissue interactions [8,9]. The oral microbiome can directly influence the composition of electrolytes, amino acids, lipids, cytokines, and pH of saliva [10,11]. Furthermore, protein markers can be altered by microbial metabolism [12]. Without consideration of the oral microbiome, it is difficult to distinguish changes in saliva composition caused by tumor progression and systemic inflammation from those associated with local dysbiosis, gingivitis, caries, mucositis, xerostomia, or impaired oral mucosal barrier function [13,14]. Thus, the microbiome is an important component in the study of saliva parameters, which can both distort the interpretation of certain markers and directly provide information about tumor–host interactions that affect salivary gland function and oral health.

A combined analysis of these data will allow us to assess the potential of using saliva for diagnosis, risk assessment, and disease monitoring, as well as understanding the biological changes associated with the cancer process.

## 2. Electrolytes

Saliva is produced primarily by the parotid, submandibular, and sublingual glands. Its electrolyte composition undergoes significant changes as it passes through the salivary ducts, where Na^+^ and Cl^−^ are predominantly reabsorbed, while K^+^ and HCO_3_^−^ are secreted into the duct lumen [15,16,17]. Unlike blood electrolytes, which are tightly regulated by renal, endocrine, and systemic feedback mechanisms, the electrolyte concentration in saliva is not maintained at a constant level. It depends on plasma composition, glandular secretion, ductal epithelial transport, and, especially, the rate of salivary flow [18,19,20,21,22,23]. Therefore, the electrolyte level in saliva should not be interpreted as a direct reflection of blood electrolyte concentrations.

In the oral cavity, electrolytes contribute to local ionic balance, mucosal hydration, buffering capacity, enamel protection, digestion, and antimicrobial protection [24]. Bicarbonate is particularly important because it neutralizes acids, helps maintain oral pH, reduces enamel demineralization, and maintains microbial balance [25,26,27]. Calcium and magnesium also have broader biological significance: Ca^2+^ is involved in immune cell signaling, and Mg^2+^ is involved in the regulation of ion channels, metabolic reactions, DNA/RNA synthesis, cell proliferation, apoptosis, and immune regulation [28,29,30,31,32]. However, changes in salivary Ca^2+^ and Mg^2+^ levels should be considered nonspecific indicators of local or systemic homeostasis disturbances rather than specific tumor markers.

Cancer may indirectly affect the electrolyte composition of saliva. Tumor-related changes in hydration status, appetite, renal electrolyte balance, endocrine regulation, stress, cachexia, medication intake, and autonomic control of salivary flow may secondarily alter levels of Na^+^, K^+^, Cl^−^, HCO_3_^−^, Ca^2+^, Mg^2+^, and phosphate [33,34,35]. Furthermore, tumors may release cytokines, acute phase mediators, and extracellular vesicles into the circulation, which may affect salivary gland epithelial cells, endothelial cells, immune cells, and oral mucosal cells [36,37]. Activation of NF-κB, MAPK, and JAK/STAT signaling pathways by cytokines such as TNF-α, IL-1β, and IL-6 can alter ion channels, transporters, aquaporins, tight junction proteins, acinar fluid secretion, ductal ion reabsorption/secretion, and epithelial permeability [38,39,40].

Studies in potentially malignant oral lesions and oral squamous cell carcinoma have shown changes in salivary Na^+^, K^+^, Ca^2+^, Mg^2+^, phosphate, zinc, copper, and iron levels, but the results are inconsistent [41,42]. In distant tumors, interpretation is even more indirect, as salivary electrolyte changes are likely to reflect systemic inflammation, altered glandular secretion, hydration status, treatment effects, and oral health rather than tumor-specific electrolyte regulation [43].

In general, salivary electrolytes should be considered as low-specificity auxiliary markers. Their levels can be strongly influenced by hydration, renal function, diet, recent food intake, vomiting, diabetes, Sjögren’s syndrome, oral infections, periodontitis, smoking, alcohol consumption, medications such as diuretics or anticholinergics, chemotherapy/radiotherapy, stress, circadian rhythm, salivary flow rate, and stimulated or unstimulated salivation [44,45]. Thus, although cancer may contribute to alterations in salivary electrolyte balance, these changes alone do not indicate malignancy and should be interpreted only in conjunction with more specific molecular, inflammatory, metabolic, and clinical data.

## 3. Amino Acids

The salivary amino acid profile may reflect tumor-associated metabolic changes, systemic inflammation, cancer cachexia, tissue catabolism, immune dysregulation, and changes in nutritional status [46]. As tumors progress, amino acids are increasingly required for cell proliferation, protein and nucleotide synthesis, regulation of redox balance, and energy metabolism [47]. At the systemic level, malignancies may also promote muscle protein breakdown, reduced nutrient intake, altered liver metabolism, and inflammatory redistribution of amino acid pools [48,49]. Therefore, changes in amino acid levels should be interpreted as part of a broader metabolic response to cancer rather than as tumor-specific markers.

Cancer-associated metabolic reprogramming can alter circulating amino acid pools. Amino acids are involved in energy metabolism, nitrogen and carbon transport, immune regulation, redox balance, and extracellular matrix remodeling [50,51]. For example, arginine may support nitric oxide production and VEGF-related angiogenic signaling [52]; Met, cysteine, glutamine, and glutamine are associated with glutathione metabolism and antioxidant defense [53,54]; and altered tryptophan metabolism may contribute to kynurenine-related immunosuppressive pathways [55,56,57]. Although these mechanisms have important biological significance, in the context of salivary diagnostics they should be considered primarily as explanations for systemic metabolic disturbances rather than as direct evidence that the measured amino acids in saliva originate from tumor tissue.

Saliva may reflect these systemic metabolic changes, as amino acids can enter the oral cavity from multiple sources, including plasma, salivary gland secretions, gingival fluid, oral mucosal transudate, immune cells, epithelial turnover, and microbial metabolism [58,59,60,61,62]. Free amino acids can enter saliva via uptake from the blood by acinar and ductal cells, selective epithelial transport, limited paracellular transport, and direct entry into primary saliva [63,64,65,66,67,68,69]. Furthermore, mixed saliva contains epithelial cells, leukocytes, microorganisms, food debris, and salivary proteins, which can undergo proteolysis and postscretory modifications in the oral cavity, further altering the measured amino acid profile [70,71,72].

In non-oral malignancies such as pancreatic, breast, lung, colon, ovarian, and prostate cancers, amino acids are not typically released directly into saliva by the tumor [73]. Instead, changes in saliva likely occur indirectly through systemic metabolic reprogramming, inflammation, cachexia-associated protein degradation, extracellular vesicles/exosomes, and altered salivary gland secretory activity [74,75,76,77,78]. These mechanisms may alter the plasma amino acid pool and influence salivary gland function, thereby altering saliva composition.

Clinical interpretation of salivary amino acids remains limited by low specificity. Salivary amino acid levels can be altered by anticancer therapy, xerostomia, mucositis, infections, periodontitis, dietary changes, medications, oral health, and salivary flow rate [79,80,81]. Thus, although salivary amino acids may provide useful information about the biochemical consequences of advanced malignancy, systemic inflammation, nutritional status, disease severity, and treatment-related changes [82,83], they should not be interpreted as independent tumor-specific diagnostic biomarkers. Instead, they are better considered as supporting components of an integrated salivary metabolic profile, along with inflammation, lipids, oxidative stress, the microbiome, and clinical data.

## 4. Lipids

**Figure 2:**
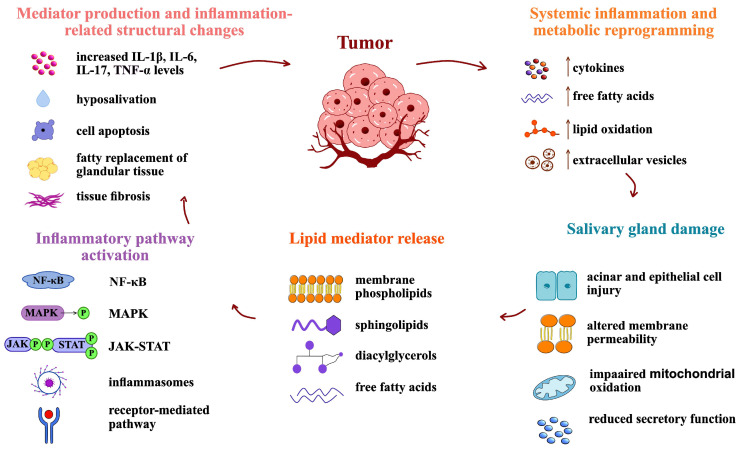
Conceptual model of the vicious circle of lipid-inflammatory remodeling.

Thus, a vicious cycle of lipid-inflammatory remodeling can develop in cancer (Figure 2). The tumor triggers a systemic inflammatory response and metabolic reprogramming, which increases circulating levels of cytokines, free fatty acids, oxidized lipids, and extracellular vesicles. These factors damage the epithelial and acinar cells of the salivary glands, disrupting membrane stability, mitochondrial β-oxidation, and secretory function. Damaged cells, in turn, release membrane phospholipids, sphingolipids, diacylglycerols, and free fatty acids, which can further activate NF-κB, MAPK, JAK-STAT, inflammasome-mediated, and receptor-mediated inflammatory pathways. This supports the production of IL-6, IL-17, TNF-⍺, IL-1β and other mediators, enhancing apoptosis, hyposalivation, fatty replacement of glandular tissue and fibrosis [84]. The lipid profile of saliva is formed by multiple sources, including salivary gland secretion, plasma-derived components, gingival fluid, extracellular vesicles, desquamated epithelial cells, membrane fragments of eukaryotic and prokaryotic cells, and products of microbial lipolytic activity [33]. Therefore, salivary lipids should be interpreted as integrated biomarkers of local and systemic lipid-related changes, rather than as direct indicators of a single pathological source. Extracellular vesicles are particularly relevant because they contain a lipid bilayer and may transport proteins, nucleic acids, and lipids, reflecting intercellular communication and potentially systemic pathological processes [85,86].

From a biomarker perspective, changes in phospholipids, sphingolipids, cholesterol, glycolipids, diacylglycerols, triacylglycerols, and free fatty acids may reflect membrane turnover, epithelial desquamation, cell damage, inflammation, microbial lipase activity, or altered salivary gland secretion [87,88,89]. In oral cancer, these changes may be more closely associated with local epithelial injury, tumor-associated inflammation, necrosis, apoptosis, and direct contact between tumor tissue and oral fluid. In contrast, in distant tumors, salivary lipid alterations should be interpreted more cautiously, because they are unlikely to result from direct tumor release into saliva.

Chronic inflammation and salivary gland dysfunction may also influence the salivary lipid profile. In inflammatory glandular diseases, prolonged inflammation may be associated with acinar damage, impaired mitochondrial metabolism, reduced secretory function, hyposalivation, ductal changes, fibrosis, and inflammatory infiltration [90,91]. Studies in primary Sjögren’s syndrome show that saliva, tears, and minor salivary glands can have altered lipidomic profiles, including changes in sphingomyelin, phosphatidylcholine, and diacylglycerols [92]. These findings support the idea that salivary lipid changes may reflect glandular remodeling and secretory dysfunction, although they are not specific to cancer.

In distant malignancies, salivary lipid changes may be linked to a systemic-local axis rather than direct tumor involvement of the oral cavity. Tumor-associated systemic inflammation, metabolic reprogramming, acute-phase mediators, lipid metabolites, and extracellular vesicles may reach the salivary glands through the bloodstream and influence epithelial, immune, and secretory activity [93]. In this context, altered salivary lipids may reflect secondary effects of systemic inflammation, changes in membrane composition, oxidative stress, altered fatty acid metabolism, hyposalivation, and possible remodeling of the salivary glands [94,95,96].

It is important to distinguish these lipid biomarkers from lipid-mediated mechanisms. Free fatty acids are not only structural membrane components and energy substrates, but may also act as signaling molecules. Saturated fatty acids, such as palmitate, have been shown in experimental models to activate NF-κB and p38 MAPK signaling in salivary gland epithelial cells, induce IL-6 production, and promote apoptosis-related pathways [97]. Fatty acids may also signal through receptors such as FFA1/GPR40 and FFA4/GPR120 on immune cells, influencing cytokine release and inflammatory pathways [98,99]. However, these mechanisms should not be directly inferred from total salivary lipid measurements unless specific fatty acid species or signaling pathways are experimentally assessed.

Different fatty acids may have opposite biological effects. Palmitic acid is generally associated with pro-inflammatory signaling, whereas oleic acid and some omega-3 fatty acids may show anti-inflammatory effects in experimental systems by reducing iNOS, COX-2, TNF-α, IL-1β, IL-6, and NF-κB/MAPK activation [100]. Short-chain fatty acids may also participate in immune regulation of the salivary glands; for example, butyrate has been associated with increased salivary flow, reduced glandular inflammation, and modulation of IL-10- and IL-17-producing B-cell populations in experimental models [79,80]. These observations suggest that not only total lipid content, but also lipid class composition and fatty acid balance may be biologically relevant.

Changes in salivary lipid composition in cancer should be interpreted as auxiliary, low-specificity biomarkers of inflammatory-metabolic and membrane changes. They can provide information about epithelial damage, salivary gland dysfunction, oxidative stress, the contribution of extracellular vesicles, microbial activity, hyposalivation, and systemic metabolic disturbances. However, changes in salivary lipid composition alone are insufficient to prove a causal relationship with lipids or to indicate malignancy. They should be analyzed in conjunction with cytokines, oxidative stress markers, amino acids, microbiome-related metabolites, salivary flow rate, oral health status, treatment history, and clinical data.

## 5. Cytokines

Cytokines from the blood can enter saliva via the salivary glands primarily through limited paracellular passage, possible vesicular/transcellular trafficking, and inflammation-induced barrier dysfunction [81]. However, in whole saliva, many cytokines are not simply filtered from serum. They often reflect a mixture of molecules from the blood, salivary gland epithelial secretions, immune cell production, gingival fluid, and oral mucosal inflammation. Therefore, saliva may reflect systemic inflammation, and salivary cytokine levels should be interpreted as an integrated oral-systemic signal rather than as a direct reflection of blood cytokine concentrations [101,102].

In paracellular leakage, cytokines can cross between epithelial cells if the tight junctions in the blood–saliva barrier (BSB) are permeable. This is usually limited because cytokines are relatively large proteins, but inflammation, radiation, Sjögren’s syndrome, infection, or epithelial injury can weaken tight junctions [103]. Recent studies of tricellulin, a tricellular tight junction protein, support the idea that salivary gland acinar junctions normally limit the penetration of macromolecules and that barrier disruption may allow larger molecules to cross more easily [104]. If transcellular vesicular transport is involved, cytokines may be taken up by the basement membrane by endocytosis and moved across epithelial cells in vesicles, then released apically into the lumen. This pathway is plausible for some proteins and immune mediators, but for most cytokines, it is less well understood than the classical salivary transport of IgA or small lipophilic molecules [105]. In receptor-mediated uptake or signaling, many cytokines bind to receptors on salivary gland epithelial cells. In this case, a cytokine from the blood may not simply “pass through” the receptors; it may activate the epithelial cell, which then secretes its own cytokines or chemokines into saliva. Salivary gland epithelial cells are now recognized as active participants in the immune process, particularly in inflammatory diseases such as Sjögren’s syndrome [106,107].

Cytokines and other protein biomarkers from the blood can potentially enter saliva at the level of the salivary glands, including near the acinar region where primary saliva is formed. This is because acinar cells produce a primary isotonic secretion that is then modified by ducts, and the salivary glands are closely connected to the vascular and interstitial space. Therefore, the most likely site of transfer of serum biomarkers into glandular secretions may be the acinar epithelium and the blood–salivary barrier, although the contribution of this mechanism should be assessed separately for each cytokine [60,108]. Ductal cells may also contribute by releasing immune mediators or altering the protein composition of the fluid, but ducts are usually considered in terms of modification of ions and electrolytes rather than passive transfer of cytokines [109]. Gingival fluid is the major non-glandular pathway for entry of substances from the blood. Cytokines contained in this fluid can mix with mixed saliva without passing through the epithelium of the salivary glands [110]. Studies comparing the composition of saliva and gingival fluid show that the cytokine profiles in these two fluids can differ significantly [111,112].

A significant proportion of salivary cytokines can be synthesized locally in the oral cavity rather than transported unchanged from the blood [113]. The main sources are acinar and ductal epithelial cells, particularly when stimulated by infection, injury-related signals, interferon’s, or autoimmune inflammation; resident and infiltrating immune cells in salivary gland tissue; oral epithelial cells, periodontal tissues, and leukocytes in gingival fluid [114]. This local synthesis explains why salivary and serum cytokine levels often correlate poorly or inconsistently. Some studies have found an association between systemic and salivary cytokines in certain diseases, but this association is compartment specific and depends on oral health, salivation rate, circadian rhythm, collection method, and local inflammation [115].

The most convincing data on the increase in cytokines in saliva were obtained in squamous cell carcinoma of the oral cavity, where the tumor lesion is in direct contact with the tissues that form the composition of the oral fluid. A wide range of cytokines and inflammatory proteins were assessed in the studies, including IL-4, IL-6, IL-10, IL-13, IL-1β, IL-1RA, IL-17A, IL-17F, IFN-γ, and TNF-⍺, hepatocyte growth factor (HGF), C-reactive protein and VEGF [116]. IL-8 turned out to be the most studied, the level of which in all the studies reviewed was significantly increased in patients with squamous cell carcinoma of the oral cavity compared to the control. Increased IL-1β, IL-17A, IL-17F, TNF-⍺, HGF, and VEGF have also been reported, some of which were associated with more advanced stages, larger tumor size, and lymph node involvement [117]. These data indicate that the salivary cytokine profile is sensitive to tumor-associated inflammation.

However, in oral cancer, the increase in cytokines can be explained predominantly by local sources, namely tumor tissue, inflammatory infiltrate, damaged epithelium, gingival fluid, vascular exudate, and cellular debris. Therefore, in distant malignancies, this mechanism should be interpreted differently. Cytokines do not enter saliva directly from the tumor site, but through the systemic “tumor–blood–salivary glands–saliva” axis. In distant tumors, tumor cells, stroma, and immune cells of the microenvironment can release IL-1β, IL-6, IL-8, IL-10, IL-17, TNF-⍺, VEGF, and extracellular vesicles into the bloodstream [118]. These mediators are able to reach the salivary glands and oral mucosa, changing vascular permeability, epithelial barrier and secretory activity of acinar and ductal cells. Through activation of JAK/STAT, NF-κB and MAPK/p38 pathways, they can increase local inflammation, disrupt tight intercellular junctions, change ion transport and secretion composition, as a result of which the cytokine profile of saliva becomes a reflection of not only local processes but also the systemic inflammatory effect of a distant tumor [119]. Once cytokines reach the basal side of salivary gland epithelial cells, three modes of their transport are possible, namely, paracellular leakage, transcellular vesicular transport, receptor-mediated uptake or signaling [120].

## 6. Tumor Markers

Tumor markers in saliva can reflect both local tumor processes in the oral cavity and systemic changes associated with tumors of distant localization. Their appearance in salivary fluid can occur due to direct release by tumor cells in oral cancer, as well as due to tissue damage, inflammation and cellular decay in the tumor area [121]. In distant malignant neoplasms, tumor-associated proteins, nucleic acids and other molecules can enter saliva through the bloodstream, salivary glands, gingival fluid and increased vascular-epithelial permeability [122]. Of particular importance are exosomes and other extracellular vesicles, which are capable of transporting tumor DNA, RNA, microRNA, proteins and lipids, providing communication between the tumor and the salivary glands. Furthermore, the tumor may alter salivary composition indirectly through systemic inflammation, immune activation, oxidative stress, and metabolic reprogramming of the host [123].

In the context of cancer, this mechanism is particularly important to explain how tumor markers located outside the oral cavity can appear in saliva in both normal and pathological conditions. Tumor markers that can normally be synthesized in small amounts in the oral cavity or saliva include: squamous cell carcinoma antigen 1 (SCCA1), squamous cell carcinoma antigen 2 (SCCA2), CYFRA 21-1, tissue polypeptide-specific antigen (TPS), CA125 (MUC16, mucin 16), CA19-9, human epididymis protein 4 (HE4), human epididymis protein 4/WAP four-disulfide core domain protein 2 (HE4/WFDC2), and CA15-3 (MUC1). The SCCA antigen (SCCA1, SCCA2) is produced by normal squamous epithelium. Normal oral mucosa can express SCCA antigens. One study identified SCCA antigen expression levels in normal and hyperplastic oral mucosa samples, not just carcinoma [124]. CYFRA 21-1 is a fragment of cytokeratin 19 derived from epithelial cells. Cytokeratins are normal structural proteins of the oral mucosa, and CYFRA 21-1 can be detected in saliva [125]. TPS is associated with cytokeratin turnover from epithelial cells; it has been studied in saliva as a marker of oral cancer and was detected at baseline levels in healthy individuals [126,127]. CA125 (MUC16) is synthesized by some normal epithelial cells of the mucous membranes of the respiratory epithelium, cornea, and conjunctiva and, accordingly, can be released into saliva. MUC16 is usually located on the apical surface of epithelial cells and performs a protective and lubricating function [128]. CA19-9 is normally synthesized in small amounts mainly by epithelial cells of the glandular-ductal type. CA19-9 is also called sialyl Lewis A. It is a carbohydrate antigen associated with mucins. It is not tumor-specific because it can be synthesized by normal epithelial cells [129,130]. HE4 is synthesized by epithelial cells of the salivary glands, particularly ductal and mucous cells. Studies of normal tissues show high expression of HE4/WFDC2 in the salivary glands, as well as in glands of the nose, sinuses, posterior tongue, and tonsils [131,132]. CA15-3 (MUC1) is normally synthesized by epithelial cells, including salivary glands [133].

Direct release of tumor-associated molecules into saliva has been shown in oral squamous cell carcinoma, where tumor tissue is in direct contact with oral fluid. Proteomic studies have revealed changes in the levels of a number of salivary proteins in patients with oral squamous cell carcinoma, including Galectin-3-binding protein (LGALS3BP), myeloid-related protein 14 (MRP14), CD59, profilin-1, catalase, defensin-1, soluble CD44, PKC antigen, CEA, CYFRA 21-1, MMP-2, MMP-11, lactate dehydrogenase (LDH), 8-hydroxy-2′-deoxyguanosine (8-OHdG), and other molecules associated with tumor growth, inflammation, apoptosis, oxidative stress, and extracellular matrix remodeling [134]. The following markers can be synthesized locally in the oral cavity by salivary gland cells, mucosal epithelium, immune cells, fibroblasts, endothelium, and tumor microenvironment cells: cytokines; chemokines; immune mediators (IL-6, IL-8); TNF-⍺; tumor necrosis factor ligand superfamily member 10 (TNFSF10); soluble Fas receptor (sFAS/TNFRSF6); soluble Fas ligand (sFasL); weak inducer of apoptosis, tumor necrosis factor-like (TWEAK, Tumor necrosis factor ligand superfamily member 12); growth and angiogenesis factors (VEGF-A, HGF, fibroblast growth factor 2/basic fibroblast growth factor (FGF-2/FGF-basic), transforming growth factor alpha (TGF-α), stem cell factor (SCF)); proteins of inflammation, innate immunity and stress response (macrophage migration inhibitory factor (MIF), myeloperoxidase, ferritin, galectin-3, chitinase-3-like protein 1 (YKL40/CHI3L1), growth differentiation factor 15 (GDF15) [135], GDF15 (Human Protein Atlas [136])); proteins of the extracellular matrix and tissue remodeling (osteopontin (OPN), osteonectin/secreted protein acidic and rich in cysteine (SPARC), periostin, osteoprotegerin (OPG), fibroblast activation protein (FAP), cathepsin D, transglutaminase 2 (TGM2)); epithelial, adhesion and tumor-associated proteins (CD44 [CD44 (Human Protein Atlas [136]], epithelial cell adhesion molecule (EpCAM), CYFRA21-1, CA15-3/MUC1, CA125/MUC16 (MUC16 (Human Protein Atlas) [136]), CEA/CEA-related cell adhesion molecule 5 (CEACAM5 (Human Protein Atlas) [136]), CA19-9, CA9, aldehyde dehydrogenase 1A1 (ALDH1A1, aldehyde dehydrogenase 1 family member A1), mesothelin, midkine, cell adhesion molecule L1/neural cell adhesion molecule L1 (NCAML1/L1CAM/CD171, Neural cell adhesion molecule L1/L1 cell adhesion molecule/cluster of differentiation 171), WNT signaling pathway inhibitor 1 Dickkopf (DKK1, Dickkopf WNT signaling pathway inhibitor 1), insulin-like growth factor binding protein 3 (IGFBP3), kallikrein-6, HE4 (WFDC2) [WFDC2 (Human Protein Atlas) [136]].

Leptin, prolactin, melanoma inhibitory activity (MIA), hepsin, DKK1, neuron-specific enolase (NSE), periostin, and tartrate-resistant acid phosphatase type 5 (TRAP5) can be detected in saliva due to both systemic entry from the bloodstream through the blood–salivary barrier and local synthesis in oral tissues. Potential local sources include salivary gland cells, mucosal epithelium, minor salivary gland cells, gingival and periodontal ligament fibroblasts, osteoclasts, inflammatory infiltrate cells, and tumor cells in oral and salivary gland lesions. TRAP5 is secreted by active osteoclasts and has been studied in gingival fluid/saliva in periodontal lesions and periapical processes [137]. NSE is expressed in dental pulp and nerve fibers and increases during pulp inflammation. NSE positivity has also been described in salivary gland tumors and rare neuroendocrine tumors of the oral cavity. A local source is possible due to neural elements and neuroendocrine cells of the oral cavity [138]. Periostin is highly associated with periodontal tissues. It is found in normal gingiva, at the epithelial-connective tissue junction, and among fibroblasts. Gingival fibroblasts can produce periostin, and its level in saliva has been studied in periodontitis [139]. DKK1 is considered a prognostic gene and a potential therapeutic target in oral squamous cell carcinoma; there are also data on DKK1 expression in tumor tissues and normal oral epithelium [140]. According to the Human Protein Atlas, hepsin expression is observed in salivary gland tissue, which allows for a local contribution of salivary tissues [Salivary Gland Tissue (Human Protein Atlas) [136]]. Also, the Human Protein Atlas reports MIA/MIA2 expression in oral mucosal lesions and in oral squamous cell carcinoma [MIA (Human Protein Atlas) [136]]. Prolactin binding has been described in the tissues of minor salivary glands, and prolactin receptors have been identified in the salivary glands. Prolactin neosynthesis by acinar cells of minor salivary glands has been reported in Sjögren’s syndrome [141]. Leptin is produced, accumulated, and secreted by the salivary glands and is also expressed in the oral mucosa [142]. In distant cancers, leptin, prolactin, MIA, hepsin, DKK1, NSE, periostin and TRAP5 can be detected in saliva due to systemic entry from the bloodstream through the hematosalivary barrier, as well as due to local production in the tissues of the oral cavity [143,144,145,146,147].

In distant cancers, circulating tumor-associated molecules, pro-inflammatory mediators, and products of systemic immune activation can potentially alter the composition of gingival fluid and then enter whole saliva. PSA (free, total) [143,148,149], AFP [150,151], β-subunit of human chorionic gonadotropin (HCGβ, beta subunit of human chorionic gonadotropin) [152,153], sex hormone-binding globulin (SHBG) [154,155] can be detected in saliva due to their entry from the systemic circulation through the hematosalivary barrier. AFP is synthesized predominantly by the liver-embryonic/germ cell marker [156]. HCGβ is synthesized by trophoblasts [157]. PSA (free, total) is classically associated with the prostate [158]. SHBG is a predominantly systemic protein, primarily of liver origin [159]. After synthesis in organ-specific tissues, markers can enter the blood and then penetrate into salivary secretions via transcellular and paracellular transport, ultrafiltration, and components of mixed saliva. However, the diagnostic value of salivary PSA (free, total), AFP, HCGβ, and potentially SHBG is limited because their levels depend not only on serum concentrations but also on the state of the salivary glands, the rate of salivary secretion, local inflammation, and protein stability in oral fluid [160]. Exosomes and other extracellular vesicles are one of the most compelling mechanisms explaining how distant tumors can influence saliva composition. Tumor cells secrete membrane vesicles containing proteins, DNA fragments, mRNA, microRNA, lipids, and other molecules that can enter the bloodstream, reach the salivary glands, and alter the composition of salivary secretions. In an experimental model of pancreatic cancer, it was shown that suppression of tumor exosomes biogenesis resulted in the disappearance of discriminant salivary transcriptome biomarkers, supporting a causal role of tumor-derived exosomes in the formation of salivary biomarker signatures in distal systemic diseases [161]. This mechanism is particularly important because it provides a biological explanation for the association between a distant tumor and changes in saliva without direct contact of the tumor with the oral cavity. Reviews indicate that tumor exosomes can transport tumor-specific mRNA, microRNA, DNA mutations, and proteins. In pancreatic and lung cancer models, it has been shown that tumor molecular cargo can travel through the circulation and be detected in saliva [76,77].

## 7. Microbiome

**Figure 3:**
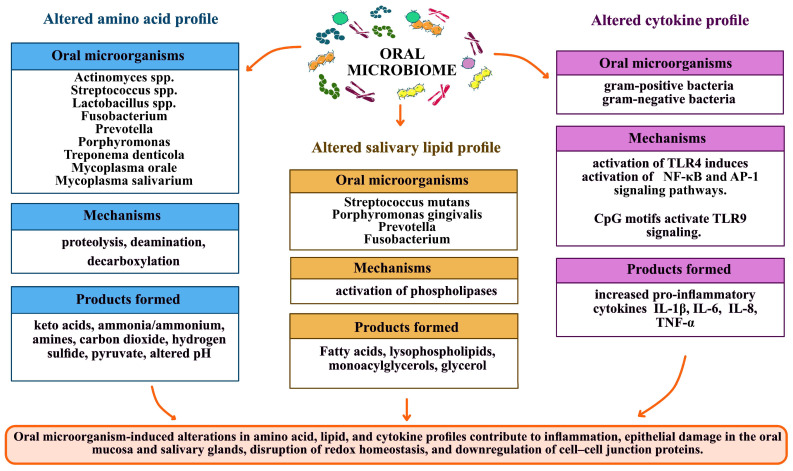
The influence of oral microorganisms on the amino acid, lipid and cytokine profile of saliva.

The oral bacterial population produces significant amounts of metabolites, including carbohydrate, lipid, and protein metabolites: short-chain fatty acids, amines, organic acids, volatile sulfur compounds, and gases [162] (Figure 3). Host enzymes and microbial proteases degrade salivary glycoproteins and amino acids; under anaerobic conditions, *Actinomyces*, *Streptococcus*, and *Lactobacillus* promote the formation of ethanol, lactic, formic, and acetic acids via the Embden-Meyerhof-Parnas pathway [163,164]. Proteolytic bacteria can increase the concentration of free amino acids, amines, and their degradation products, which are associated with inflammation and tissue damage.

Inflammation increases cellular breakdown, phospholipase activity, and the formation of bacterial lipid metabolites. Nitrate-reducing bacteria, using acids as a carbon source, can increase pH by producing ammonia during the metabolism of arginine, urea, and nitrates. In particular, nitrate can be reduced to ammonia via the bacterial dissimilatory nitrate reduction to ammonium (DNRA) pathway [165], which helps maintain acid-base balance in collaboration with acid-forming bacteria [166,167]. The oral microbiome is capable of influencing the composition of saliva directly, through the production and degradation of amino acids, lipids, organic acids and nitrogen-containing compounds, and indirectly, through inflammation, cytokine response, changes in the permeability of epithelial barriers and possible impact on transport processes in the ductal and glandular epithelium of the salivary glands [168,169]. Microbial metabolism may also indirectly influence the electrolyte composition of saliva. Bacterial acid production, ammonia generation through arginine and nitrate metabolism, and inflammation-associated changes in epithelial transport and tight junction permeability can modify local pH, bicarbonate buffering, and ion transport across the salivary gland ductal epithelium.

Taken together, microbial activity may influence all major biomarker classes discussed in this review. Bacterial metabolism contributes to alterations in amino acid and lipid profiles, microbial products activate inflammatory signaling leading to cytokine production, while inflammation-induced barrier dysfunction and altered salivary gland function may subsequently modify electrolyte composition and the transport of circulating tumor-associated biomarkers into saliva. Therefore, interpretation of salivary biomarkers should consider the oral microbiome as a potential upstream regulator rather than merely a confounding variable.

Modern studies of the oral microbiome usually rely on sequencing-based approaches. The most used method is 16S rRNA gene amplicon sequencing, which allows taxonomic profiling of bacterial communities and comparison of relative abundance between groups. However, this approach provides limited species-level resolution and does not directly measure functional activity [170,171]. Shotgun metagenomic sequencing provides deeper taxonomic and functional information, including the potential metabolic pathways of oral microbial communities [172]. In some studies, metatranscriptomic, metaproteomic, or metabolomic approaches may be combined with sequencing data to distinguish between the mere presence of microorganisms and their active metabolic contribution to saliva composition [173,174]. Therefore, interpretation of salivary microbiome data should consider not only which taxa are present, but also which metabolic pathways are predicted or experimentally detected [174].

An important methodological limitation of salivary microbiome studies is the risk of contamination and technical bias. Saliva is a low-biomass and environmentally exposed biological fluid; therefore, microbial DNA from sampling devices, reagents, laboratory surfaces, extraction kits, and sequencing procedures may influence the observed microbial profile [175,176,177,178]. In addition, oral sampling is affected by food intake, oral hygiene, smoking, circadian rhythm, dental status, periodontal inflammation, xerostomia, recent antibiotic use, anticancer treatment, and storage conditions [179,180,181,182]. For this reason, microbiome studies should include negative controls, standardized sampling protocols, careful DNA extraction procedures, and bioinformatic filtering of likely contaminants [175,176,177,178,180]. Without these precautions, it may be difficult to distinguish true cancer-associated or treatment-associated microbial changes from pre-analytical or analytical artifacts [175,176,177,178].

The amino acid profile of saliva is altered through proteolysis, deamination, and decarboxylation [183]. These microorganisms include the following groups of bacteria: saccharolytic (*Streptococcus*, *Actinomyces*, *Lactobacillus*) [184], proteolytic (*Fusobacterium*, *Prevotella*, *Porphyromonas*) [165], spirochetes (oral spirochetes, primarily *Treponema denticola*) [185], and mycoplasmas (*Mycoplasma orale*, *Mycoplasma salivarium*) [186,187]. Microbial proteases break down salivary proteins and glycoproteins, forming free amino acids. Further, amino acids can undergo deamination, with the formation of α-keto acids and ammonia. This leads to an increase in the concentration of ammonia/ammonium and changes the pH [188,189]. Decarboxylation produces biogenic amines with the release of CO_2_; for example, histidine is converted to histamine, lysine to cadaverine, and ornithine to putrescine. Sulfur-containing amino acids serve as substrates for the formation of volatile sulfur compounds: the metabolism of cysteine produces hydrogen sulfide, ammonia, and pyruvate, while the metabolism of methionine produces methanethiol, α-ketobutyrate, and ammonia [190,191,192]. These products may contribute to inflammation, the development of halitosis, epithelial damage, and changes in the redox balance of saliva.

The lipid profile can be altered by bacterial and cellular phospholipases [193]. Such bacteria include *Streptococcus mutans*, *Porphyromonas gingivalis*, and representatives of the genera *Prevotella* and *Fusobacterium*. The oral microbiome is capable of breaking down lipids using lipases [194] and using the resulting fatty acids and glycerol as a source of carbon. For example, phospholipids, under the action of phospholipases, form lysophospholipids and free fatty acids [195]. Diacylglycerols are hydrolyzed to form monoacylglycerols and free fatty acids. Inflammation, cellular damage, and the activity of microbial enzymes in saliva can increase the level of membrane lipid derivatives, plasmalogens, diacylglycerols, and free fatty acids [196]. It is precisely these lipid derivatives that have been described as part of a salivary metabolite signature reflecting host–microbiome interactions in experimental gingivitis [197].

Cytokine composition and barrier permeability are altered through immunobiochemical reactions recognizing microbial molecules. Bacterial components such as lipopolysaccharide from Gram-negative bacteria, lipoteichoic acids from Gram-positive bacteria, peptidoglycan, and bacterial DNA activate innate immune receptors: lipopolysaccharide activates Toll-like receptors 4 (TLR 4), which in turn activates NF-κB/activator protein 1 (AP-1). This leads to the activation of IL-1β, IL-6, IL-8, and TNF-α [198]. Lipoteichoic acid and peptidoglycan activate TLR2, which triggers the activation of NF-κB and leads to the potentiation of pro-inflammatory cytokines [199]. Bacterial DNA CpG motifs (CpG, Cytosine–phosphate–guanine) can activate TLR9, leading to an inflammatory response. Cytokines and bacterial proteases then activate matrix metalloproteinases [200], which weakens intercellular contacts and the extracellular matrix. Simultaneously, inflammatory mediators can reduce the expression or disrupt the distribution of tight junction proteins, including occludin, claudins, and ZO-1 (Zonula occludens-1), which increases epithelial permeability and facilitates the entry of serum proteins, ions, cytokines, and tissue damage products into mixed saliva [201].

A distant cancer process can act as a feedback axis, altering the oral ecosystem even without primary damage to the salivary glands or oral mucosa. For example, a different salivary microbiota composition has been described in patients with non-small cell lung cancer compared to healthy individuals. Moreover, salivary dysbiosis has been associated with systemic inflammatory markers and predicted changes in microbial metabolism, including amino acid, carbohydrate, and lipid metabolism pathways. This suggests that extraoral cancer may be associated with a restructuring of the salivary microbiome through systemic inflammation, immune dysregulation, and metabolic shifts [202]. However, such associations should be interpreted carefully. In most clinical studies, it remains difficult to determine whether dysbiosis is a causal contributor to carcinogenesis, a consequence of the tumor-induced systemic inflammatory state, or a secondary effect of treatment, oral complications, altered nutrition, xerostomia, antibiotics, and changes in hygiene behavior [203,204,205,206]. Therefore, salivary dysbiosis should not automatically be interpreted as a primary cause of cancer. It may also represent a consequence of systemic inflammation, immune dysregulation, altered saliva composition, or therapeutic intervention [207]. Additionally, anticancer therapy enhances this effect. Chemotherapy, radiation therapy, and hematopoietic stem cell transplantation are associated with significant changes in oral microbial communities [208]. Common clinical sequelae include mucositis, infections, salivary gland dysfunction, xerostomia, taste disturbances, pain, dehydration, and nutritional disturbances. For example, in the treatment of head and neck tumors, radiotherapy damages the salivary glands and causes hyposalivation/xerostomia, and the dry, less buffered, and more inflammatory oral environment promotes the growth of opportunistic and inflammatory microorganisms [205,207,209]. Therefore, in oncological settings, salivary dysbiosis may reflect not only the presence of cancer, but also the cumulative biological impact of therapy, immune suppression, epithelial injury, and changes in the physicochemical properties of saliva. This is especially relevant for patients receiving chemotherapy or radiotherapy, where mucositis, hyposalivation, xerostomia, altered pH, reduced buffering capacity, and opportunistic infections may independently reshape the oral microbial community. Tumor-derived extracellular vesicles may further contribute to this process by transporting proteins, nucleic acids, lipids, and signaling molecules capable of modifying salivary gland function and oral immune responses [210,211,212,213].

This creates a vicious cycle: the tumor, along with associated therapy, causes systemic inflammation, immune dysregulation, cachexia, drug overload, and damage to the salivary glands. This leads to changes in the volume and composition of saliva, dysbiosis, local inflammation, and increased permeability of epithelial and glandular barriers [209], including decreased salivary flow, reduced mechanical cleansing and buffering capacity, and shifts in pH and electrolyte composition, which alters the conditions for bacterial growth. Against this background, the cytokine and immunomediator components acquire particular significance. In addition to IL-1β, IL-6, IL-8, and TNF-α, IL-17A, IL-22, IL-23, IFN-γ, IL-10, transforming growth factor beta (TGF-β), monocyte chemoattractant protein-1 (MCP-1), regulated upon activation, normal T cell expressed and secreted (RANTES), interferon gamma-induced protein 10 (IP-10), granulocyte colony-stimulating factor (G-CSF), granulocyte-macrophage colony-stimulating factor (GM-CSF), and VEGF may be involved in maintaining the inflammatory microenvironment [214,215,216,217,218,219,220,221,222]. These molecules regulate the recruitment of neutrophils, monocytes, macrophages and lymphocytes, epithelial cell activation, vascular permeability, tissue remodeling and mucosal repair. Additionally, the levels of complement components C3, C3a [223] and C5a [224], secretory IgA, IgG, IgM [225], as well as antimicrobial proteins and peptides—lysozyme, lactoferrin [226], histatins [227], α- and β-defensins, antimicrobial peptide cathelicidin LL-37 (LL-37, Human cathelicidin antimicrobial peptide LL-37) [228], secretory leukocyte protease inhibitor (SLPI) [229] and calprotectin S100A8/A9 [230,231] can change in saliva. These molecules directly influence microbial adhesion, bacterial growth, neutrophil inflammation, barrier permeability, and oral microbiome composition [232,233]. Furthermore, bacterial proteases, lipopolysaccharides, organic acids, amines, and sulfur compounds damage tight junction proteins and increase permeability of the oral mucosa and ductal epithelium. Epithelial damage and inflammation create substrates for microbial growth, and dysbiotic microbiota, in turn, supports inflammatory amplification and delays healing [234,235].

Thus, the oral microbiome in cancer patients should be considered not only as a marker of saliva status, but also as an active link in a self-sustaining axis: the oncological process alters the salivary environment, altered saliva restructures the microbiome, and dysbiosis perpetuates inflammation, barrier dysfunction, and further changes in the electrolyte, lipid, amino acid, and cytokine composition of saliva. At the same time, current evidence does not always allow dysbiosis to be classified strictly as a cause or a consequence of cancer. In many cases, it is more accurate to view it as a dynamic and context-dependent process shaped by tumor biology, systemic inflammation, local oral conditions, and therapeutic interventions.

## 8. Preliminary Conclusions on the Most Promising Candidates for Salivary Biomarkers and Priority Areas for Future Research

Despite significant progress in salivary biomarker research, only a limited number of candidates currently demonstrate sufficient biological plausibility, reproducibility, and consistency of study results to be considered particularly promising for future clinical application. Based on the data presented in Table 1 and Table 2, the most promising biomarkers can be divided into several functional categories.

Inflammatory biomarkers are among the most mature candidates. IL-8 demonstrates more convincing evidence of efficacy, particularly in oral squamous cell carcinoma (OSCC), with consistent associations identified in independent studies. IL-6, TNF-α, IL-1β, and VEGF also demonstrate relatively strong evidence of efficacy and reflect key inflammatory, angiogenic, and tumor–host interaction pathways. These cytokines are well-studied biologically and are among the most reproducible salivary biomarkers currently available. Among traditional tumor-associated proteins, CYFRA 21-1, CEA, SCCA1/SCCA2, HE4, CA125 (MUC16), and CA15-3 (MUC1) appear to be the most promising candidates. However, their diagnostic performance when assessed individually remains insufficient for routine clinical use [9,15,35,121].

Several biomarkers represent particularly attractive avenues for future research. These include exosomal RNA, CD44, EpCAM, MMP-2/MMP-11, CA9, DKK1, ALDH1A1, and mesothelin, all of which are associated with tumor invasion, epithelial plasticity, hypoxia, extracellular matrix remodeling, or cancer stem cell biology. Although available data are still limited, these biomarkers may provide greater biological specificity than traditional inflammatory markers. Similarly, 8-OHdG and LDH can serve as useful indicators of oxidative stress and tissue damage, although their lack of specificity limits their independent diagnostic value [8,40,85,86].

Biochemical signatures, rather than individual biomarkers—such as electrolytes, amino acid profiles, lipid classes, and free fatty acids—should not be considered isolated diagnostic biomarkers. Instead, they are more appropriately considered as components of multidimensional metabolic signatures reflecting salivary gland function, epithelial integrity, microbiome activity, and systemic metabolic changes. Therefore, their greatest potential will lie in combination with inflammatory and tumor-specific biomarkers, rather than as stand-alone diagnostic markers [2,13,43,73].

In terms of priorities for future research, the following can be highlighted. First, future research should move beyond the assessment of individual biomarkers to multimarker panels integrating inflammatory mediators, tumor-associated proteins, metabolites, and extracellular vesicle contents. Second, greater attention should be paid to understanding the biological origins of salivary biomarkers, distinguishing between molecules derived from direct tumor rejection, systemic circulation, salivary gland secretion, epithelial barrier dysfunction, gingival fluid, and the oral microbiota. Third, longitudinal studies are needed to distinguish biomarkers reflecting early tumor development, treatment response, disease recurrence, and treatment-induced changes. Fourth, standardized protocols for microbiome collection, processing, analysis, and biomarker quantification remain crucial to improve the reproducibility of results between studies [2,15,35,60].

Future studies should further integrate multi-omics approaches, combining proteomics, metabolomics, lipidomics, transcriptomics, exosomal composition, and microbiome profiling with clinical variables and machine learning methods to improve diagnostic performance and biological interpretation.

## 9. Conclusions

**Figure 4:**
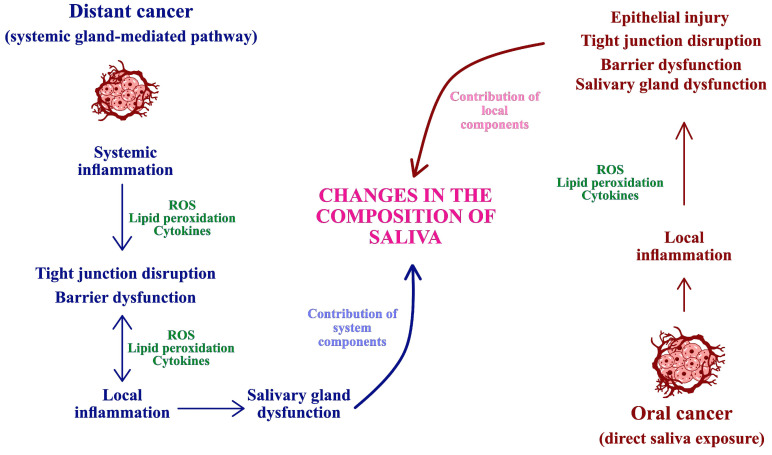
Direct and indirect pathways linking cancer to salivary alterations.

Various metabolites can enter saliva through salivary gland secretion, blood transport, gingival fluid, extracellular vesicles, epithelial desquamation, microbiota activity, and local production by oral cells. In malignant tumors not localized in the oral cavity, changes in saliva composition are more often associated not with direct metabolites entering the oral fluid from tumor tissue, but with systemic inflammation, metabolic reprogramming, tumor-derived exosomes, cachexia, oxidative stress, and secondary changes in salivary gland function. The oral microbiome also influences saliva composition by participating in the breakdown of proteins, glycoproteins, amino acids, and lipids, activating the innate immune response, enhancing cytokine production, and potentially increasing the permeability of epithelial barriers. Therefore, salivary biomarkers should be interpreted as an integral signal of the tumor–blood–salivary glands–microbiome–oral cavity axis, and their diagnostic value is most fully realized as part of multimarker panels after standardization of sample collection, accounting for variability, and clinical validation. Salivary markers may have the greatest practical value for screening at-risk groups, monitoring treatment response, early detection of adverse dynamics, and personalized assessment of systemic inflammatory stress in cancer patients (Figure 4).

Pathology, including tumor burden, can alter the composition of saliva, and localized disorders in the oral cavity can further exacerbate the inflammatory environment, impair nutrition, therapy tolerance, immune status, and the patient’s overall well-being. In the long term, pro-inflammatory cytokines and microbial products can enter the systemic circulation, creating a state of low-grade systemic inflammation. Such immune stress can contribute to the development or worsening of autoimmune diseases, endothelial dysfunction, cardiovascular disease, and metabolic disorders.

## Figures and Tables

**Table 1:** Current evidence supporting salivary biomarker classes and biological pathways associated with cancer.

Biomarker/Pathway	Oral Cancer Evidence	Distant Cancer Evidence	Clinical Maturity
Electrolytes	Weak	Weak	Not diagnostic
**Total amino acids/** **α** **-AAs**	Weak	Moderate/uncertain	Not diagnostic
**Arginine—NO-VEGF pathway**	Moderate	Emerging	Research
**Glutamine/Glutamate/Aspartate metabolism**	Moderate	Moderate	Research
**TCA cycle amino acid input**	Moderate	Moderate	Research
**Redox-related amino acids: Cys, Met, Gln, Glu**	Moderate	Moderate	Research
**Tryptophan–kynurenine pathway**	Emerging/moderate	Moderate	Research/experimental
**BCAA metabolism: Val, Leu, Ile**	Emerging	Emerging/moderate	Research
**Phenylalanine/Tyrosine metabolism**	Emerging	Emerging	Experimental
**Proline/Histidine and ROS/H_2_O_2_ regulation**	Emerging	Emerging	Experimental
**FTIR lipid CH_2_/CH_3_ profile: 1396, 1458, 2853, 2923, 2957 cm^−1^**	Moderate	Emerging	Research
**CH_2_/CH_3_ ratios: 2923/2957 and 1458/1396**	Moderate	Emerging	Research
**Phospholipids: phosphatidylcholine, phosphatidylethanolamine, etc.**	Moderate	Emerging	Research
**Sphingolipids/sphingomyelin**	Moderate	Emerging/moderate	Research
**Cholesterol/membrane rigidity pathway**	Emerging/moderate	Emerging	Research
**Diacylglycerols and triacylglycerols**	Emerging	Emerging/moderate	Research
**Free fatty acids**	Moderate	Moderate	Research
**Saturated fatty acids/palmitate—NF—κB—MAPK pathway**	Moderate	Emerging/moderate	Research
**Oleic acid and omega-3 fatty acid anti-inflammatory pathway**	Emerging	Emerging	Experimental
**FFA receptors: FFA1/GPR40 and FFA4/GPR120**	Emerging	Emerging	Experimental
**IL-8**	Strong	Moderate/uncertain	Research
**IL-6/JAK–STAT3 pathway**	Moderate/strong	Moderate/uncertain	Research
**TNF-α/NF-κB pathway**	Moderate/strong	Moderate	Research
**IL-1** **β** **/inflammasome-associated inflammation**	Moderate/strong	Emerging/moderate	Research
**IL-10/immunoregulatory pathway**	Moderate	Moderate/uncertain	Research
**IL-17A/IL-17F pathway**	Moderate	Emerging	Research
IFN-γ/Th1–cytotoxic immunity	Emerging/moderate	Emerging/moderate	Research
IFN-α/type I interferon response	Emerging	Emerging	Experimental
VEGF/angiogenic inflammatory pathway	Moderate/strong	Moderate	Research
HGF/tissue remodeling pathway	Moderate	Emerging	Research
CRP/acute-phase inflammatory signal	Emerging/moderate	Moderate	Research
Tricellulin/tight junction barrier pathway	Emerging	Emerging	Experimental
Transcellular/vesicular cytokine transport	Emerging	Emerging	Experimental
Receptor-mediated epithelial signaling	Moderate	Moderate	Research
JAK/STAT signaling	Moderate	Moderate	Research
NF-κB signaling	Strong	Moderate/ Strong	Research
MAPK/p38 signaling	Moderate	Moderate	Research
CEA	Moderate	Emerging/uncertain	Research
CA15-3/MUC1	Emerging/moderate	Moderate/uncertain	Research
CA125/MUC16	Emerging	Moderate/uncertain	Research
CA19-9/sialyl Lewis A	Emerging	Moderate/uncertain	Research
CYFRA 21-1/cytokeratin 19 fragment	Moderate	Emerging/uncertain	Research
TPS/cytokeratin turnover	Emerging/moderate	Emerging	Research
SCCA1/SCCA2	Moderate	Weak/emerging	Research
HE4/WFDC2	Emerging	Moderate/uncertain	Research
CD44/epithelial adhesion pathway	Moderate/strong	Emerging	Research
EpCAM/epithelial tumor-cell marker	Emerging/moderate	Emerging	Research
MMP-2/MMP-11/matrix remodeling	Moderate	Emerging	Research
HGF/tissue remodeling and invasion	Moderate	Emerging	Research
FGF-2, TGF-α, SCF growth-factor pathway	Emerging/moderate	Emerging	Research
LDH/tissue damage and glycolytic metabolism	Moderate	Emerging/moderate	Research (nonspecific)
8-OHdG/oxidative DNA damage	Moderate	Emerging/moderate	Research
CA9/hypoxia-associated pathway	Emerging/moderate	Emerging	Research
ALDH1A1/cancer stem-like phenotype	Emerging	Emerging	Experimental/research
Mesothelin	Emerging	Moderate/uncertain	Research
DKK1/WNT signaling pathway	Emerging/moderate	Emerging/moderate	Research
Periostin/stromal and periodontal remodeling	Moderate but confounded	Emerging	Research (strong periodontal confounder)
NSE/neural-neuroendocrine marker	Emerging	Emerging/uncertain	Research (nonspecific)
TRAP5/osteoclast activity	Emerging	Emerging	Research (periodontal/bone confounder)
Leptin	Emerging/moderate	Emerging/moderate	Research (metabolic confounder)
Prolactin	Emerging	Emerging/moderate	Research (systemic and glandular source)
PSA (free/total)	Not relevant except systemic transfer	Moderate/uncertain	Research (limited diagnostic value in saliva)
AFP	Not relevant except systemic transfer	Emerging/moderate	Research (limited diagnostic value in saliva)
HCGβ	Not relevant except systemic transfer	Emerging/moderate	Research (limited diagnostic value in saliva)
Exosomal RNA	Emerging	Emerging	Experimental

**Table 2:** Current status of salivary cancer biomarkers.

Biomarker	Cancer Type	Biological Source	Evidence Level	Clinical Readiness
**Electrolytes (Na** **^+^** **, K^+^, Cl^−^, HCO_3_^−^)**	Multiple	Salivary glands, ductal transport, plasma transudate	Weak	Not diagnostic
**Total amino** **acids/** **α** **-amino acids**	Multiple	Salivary glands, epithelial turnover, microbiome, plasma	Weak–moderate	Not diagnostic
Free fatty acids	Multiple	Cell membranes, salivary glands, microbiome	Moderate	Research
IL-1β	Oral SCC, multiple	Immune cells, epithelial cells	Moderate–strong	Research
IL-6	Oral SCC, breast, ovarian	Immune cells, salivary glands	Moderate–strong	Research
IL-8	Oral SCC	Epithelial cells, neutrophils, gingival crevicular fluid	Strong	Research
IL-10	Multiple	Immune cells	Moderate	Research
IL-17A	Oral SCC, multiple	Th17 lymphocytes	Moderate	Research
TNF-α	Multiple	Macrophages, neutrophils, epithelial cells	Moderate–strong	Research
IFN-γ	Multiple	T lymphocytes, NK cells	Emerging–moderate	Research
VEGF	Oral SCC, multiple	Epithelial cells, endothelial cells, inflammatory cells	Moderate–strong	Research
HGF	Oral SCC, multiple	Fibroblasts, stromal cells, salivary glands	Moderate	Research
CRP	Multiple	Serum transudate	Emerging–moderate	Research
CEA	GI, lung, oral	Epithelial cells, serum transudate	Moderate	Research
CA15-3 (MUC1)	Breast	Epithelial cells, serum transudate	Emerging–moderate	Research
CA125 (MUC16)	Ovarian	Serum transudate, salivary glands, epithelium	Emerging	Research
CA19-9 (Sialyl Lewis A)	Pancreatic, GI	Serum transudate, glandular secretion	Emerging	Research
HE4 (WFDC2)	Ovarian	Serum, glandular secretion	Emerging	Research
CYFRA 21-1	Oral SCC	Epithelial turnover	Moderate	Research
TPS	Multiple epithelial cancers	Cytokeratin turnover	Emerging–moderate	Research
SCCA1/SCCA2	Oral SCC	Squamous epithelium	Moderate	Research
CD44	Oral SCC	Tumor epithelium, extracellular vesicles	Moderate–strong	Research
EpCAM	Oral SCC	Tumor epithelial cells	Emerging–moderate	Research
MMP-2/MMP-11	Oral SCC	Stromal cells, inflammatory cells	Moderate	Research
FGF-2	Multiple	Stromal cells, salivary glands	Emerging–moderate	Research
TGF-α	Multiple	Epithelial cells	Emerging–moderate	Research
SCF	Multiple	Stromal cells	Emerging	Research
LDH	Multiple	Damaged epithelial cells	Moderate	Research (nonspecific)
8-OHdG	Multiple	Oxidative DNA damage	Moderate	Research
CA9	Multiple	Hypoxic tumor cells	Emerging–moderate	Research
ALDH1A1	Multiple	Cancer stem-like cells	Emerging	Experimental/Research
Mesothelin	Ovarian, mesothelioma	Tumor cells, serum	Emerging	Research
DKK1	Multiple	Tumor cells	Emerging–moderate	Research
Periostin	Oral SCC	Stromal cells, periodontal tissues	Moderate (confounded)	Research
NSE	Neuroendocrine tumors	Neuroendocrine cells	Emerging	Research (nonspecific)
TRAP5	Bone-related tumors	Osteoclasts	Emerging	Research (bone/periodontal confounder)
Leptin	Multiple	Adipose tissue, salivary glands	Emerging–moderate	Research
Prolactin	Breast, multiple	Pituitary, salivary glands	Emerging	Research
PSA (free/total)	Prostate	Serum transudate	Moderate (systemic transfer)	Research (limited diagnostic value in saliva)
AFP	Hepatocellular carcinoma	Serum transudate	Emerging	Research (limited diagnostic value in saliva)
β-hCG	Germ-cell tumors	Serum transudate	Emerging	Research (limited diagnostic value in saliva)
Exosomal RNA	Multiple	Tumor-derived extracellular vesicles	Emerging	Experimental

## Data Availability

No new data were created or analyzed in this study. Data sharing is not applicable to this article.

## References

[1] Gröschl M., Rauh M., Wagner R., Neuhuber W., Metzler M., Tamgüney G., Zenk J., Schoof E., Dörr H.G., Blum W.F. (2001). Identification of leptin in human saliva. J. Clin. Endocrinol. Metab..

[2] Padalkar P., Yadadi S.S., Vivekanandan G., Shetty S.R., Andhare M., Pashine A., Vinay V., Desai V., Shetty R.M. (2025). Salivary periostin levels as a non-invasive biomarker and their clinical correlates among healthy and periodontitis patients—A cross-sectional analytical study. Front. Dent. Med..

[3] Al-Rawi N.H., Atiyah K.M. (2009). Salivary neuron specific enolase: An indicator for neuronal damage in patients with ischemic stroke and stroke-prone patients. Clin. Chem. Lab. Med..

[4] Karataş A., Ömercikoğlu Z., Öz B., Dağlı A.F., Çatak O., Erman F., Şahin K., Gözel N., Koca S.S. (2021). Wnt signaling pathway activities may be altered in primary Sjogren’s syndrome. Turk. J. Med. Sci..

[5] Lindell S.G., Suomi S.J., Shoaf S.E., Linnoila M., Higley J.D. (1999). Salivary prolactin as a marker for central serotonin turnover. Biol. Psychiatry.

[6] Elgamal A.A., Ectors N.L., Sunardhi-Widyaputra S., Van Poppel H.P., Van Damme B.J., Baert L.V. (1996). Detection of prostate specific antigen in pancreas and salivary glands: A potential impact on prostate cancer overestimation. J. Urol..

[7] Shiiki N., Tokuyama S., Sato C., Kondo Y., Saruta J., Mori Y., Shiiki K., Miyoshi Y., Tsukinoki K. (2011). Association between saliva PSA and serum PSA in conditions with prostate adenocarcinoma. Biomarkers.

[8] Turan T., Demir S., Aybek H., Atahan O., Tuncay O.L., Aybek Z. (2000). Free and total prostate-specific antigen levels in saliva and the comparison with serum levels in men. Eur. Urol..

[9] Yio X.Y., Jiang J., Yin F.Z., Ruan K.H. (1992). Highly sensitive sandwich enzyme immunoassay for alpha-fetoprotein in human saliva. Ann. Clin. Biochem..

[10] Recker E.N., Brogden K.A., Avila-Ortiz G., Fischer C.L., Pagan-Rivera K., Dawson D.V., Smith K.M., Elangovan S. (2015). Novel biomarkers of periodontitis and/or obesity in saliva—An exploratory analysis. Arch. Oral Biol..

[11] Mahajan M., Belgaumi U.I., Baad R., Vibhute N., Kadashetti V., Bommanavar S., Kamate W. (2019). Salivary Human Chorionic Gonadotropin as a Novel Biomarker for Early Detection of Pregnancy: A Pilot Study. Gynecol. Minim. Invasive Ther..

[12] Sireesha D., Reginald B.A., Reddy B.S., Samatha M. (2021). Expression of human chorionic gonadotropin-β in tissue specimens, saliva and urine of oral squamous cell carcinoma patients. J. Oral Maxillofac. Pathol..

[13] Selby C., Lobb P.A., Jeffcoate W.J. (1988). Salivary albumin and sex hormone binding globulin (SHBG): Concentration and origin. Steroids.

[14] Hammond G.L., Langley M.S. (1986). Identification and measurement of sex hormone binding globulin (SHBG) and corticosteroid binding globulin (CBG) in human saliva. Acta Endocrinol..

[15] Hanif H., Ali M.J., Susheela A.T., Khan I.W., Luna-Cuadros M.A., Khan M.M., Lau D.T. (2022). Update on the applications and limitations of alpha-fetoprotein for hepatocellular carcinoma. World J. Gastroenterol..

[16] Ogino M.H., Tadi P. (2026). Physiology, Chorionic Gonadotropin. StatPearls.

[17] Balk S.P., Ko Y.-J., Bubley G.J. (2003). Biology of prostate-specific antigen. J. Clin. Oncol..

[18] Pugeat M., Nader N., Hogeveen K., Raverot G., Déchaud H., Grenot C. (2010). Sex hormone-binding globulin gene expression in the liver: Drugs and the metabolic syndrome. Mol. Cell. Endocrinol..

[19] Ayatollahi H., Darabi Mahboub M.R., Mohammadian N., Parizadeh M.R., Kianoosh T., Khabbaz Khoob M., Kamalian F. (2007). Ratios of free to total prostate-specific antigen and total prostate specific antigen to protein concentrations in saliva and serum of healthy men. Urol. J..

[20] Nonaka T., Wong D.T.W. (2023). Saliva diagnostics: Salivaomics, saliva exosomics, and saliva liquid biopsy. J. Am. Dent. Assoc..

[21] Mai S., Mauger M.T., Niu L.N., Barnes J.B., Kao S., Bergeron B.E., Ling J.Q., Tay F.R. (2017). Potential applications of antimicrobial peptides and their mimics in combating caries and pulpal infections. Acta Biomater..

[22] Rosier B.T., Moya-Gonzalvez E.M., Corell-Escuin P., Mira A. (2020). Isolation and Characterization of Nitrate-Reducing Bacteria as Potential Probiotics for Oral and Systemic Health. Front. Microbiol..

[23] Takahashi N. (2015). Oral Microbiome Metabolism: From “Who Are They?” to “What Are They Doing?”. J. Dent. Res..

[24] Efenberger M., Agier J., Pawłowska E., Brzezińska-Błaszczyk E. (2015). Archaea prevalence in inflamed pulp tissues. Cent. Eur. J. Immunol..

[25] Kistler J.O., Booth V., Bradshaw D.J., Wade W.G. (2013). Bacterial community development in experimental gingivitis. PLoS ONE.

[26] Abusleme L., Hoare A., Hong B.Y., Diaz P.I. (2021). Microbial signatures of health, gingivitis, and periodontitis. Periodontology.

[27] Lin D., Yang L., Wen L., Lu H., Chen Q., Wang Z. (2021). Crosstalk between the oral microbiota, mucosal immunity, and the epithelial barrier regulates oral mucosal disease pathogenesis. Mucosal Immunol..

[28] Takahashi N., Sulijaya B., Yamada-Hara M., Tsuzuno T., Tabeta K., Yamazaki K. (2019). Gingival epithelial barrier: Regulation by beneficial and harmful microbes. Tissue Barriers.

[29] Lin Y., Liang X., Li Z., Gong T., Ren B., Li Y., Peng X. (2024). Omics for deciphering oral microecology. Int. J. Oral Sci..

[30] Knight R., Vrbanac A., Taylor B.C., Aksenov A., Callewaert C., Debelius J., Gonzalez A., Kosciolek T., McCall L.I., McDonald D. (2018). Best practices for analysing microbiomes. Nat. Rev. Microbiol..

[31] Hillmann B., Al-Ghalith G.A., Shields-Cutler R.R., Zhu Q., Gohl D.M., Beckman K.B., Knight R., Knights D. (2018). Evaluating the Information Content of Shallow Shotgun Metagenomics. mSystems.

[32] Aguiar-Pulido V., Huang W., Suarez-Ulloa V., Cickovski T., Mathee K., Narasimhan G. (2016). Metagenomics, Metatranscriptomics, and Metabolomics Approaches for Microbiome Analysis. Evol. Bioinform. Online.

[33] Baker J.L. (2023). Illuminating the oral microbiome and its host interactions: Recent advancements in omics and bioinformatics technologies in the context of oral microbiome research. FEMS Microbiol. Rev..

[34] Salter S.J., Cox M.J., Turek E.M., Calus S.T., Cookson W.O., Moffatt M.F., Turner P., Parkhill J., Loman N.J., Walker A.W. (2014). Reagent and laboratory contamination can critically impact sequence-based microbiome analyses. BMC Biol..

[35] Fierer N., Leung P.M., Lappan R., Eisenhofer R., Ricci F., Holland S.I., Dragone N., Blackall L.L., Dong X., Dorador C. (2025). Guidelines for preventing and reporting contamination in low-biomass microbiome studies. Nat. Microbiol..

[36] Davis N.M., Proctor D.M., Holmes S.P., Relman D.A., Callahan B.J. (2018). Simple statistical identification and removal of contaminant sequences in marker-gene and metagenomics data. Microbiome.

[37] Lu H., Zou P., Zhang Y., Zhang Q., Chen Z., Chen F. (2022). The sampling strategy of oral microbiome. Imeta.

[38] Yano Y., Hua X., Wan Y., Suman S., Zhu B., Dagnall C.L., Hutchinson A., Jones K., Hicks B.D., Shi J. (2020). Comparison of Oral Microbiota Collected Using Multiple Methods and Recommendations for New Epidemiologic Studies. mSystems.

[39] Lim Y., Totsika M., Morrison M., Punyadeera C. (2017). The saliva microbiome profiles are minimally affected by collection method or DNA extraction protocols. Sci. Rep..

[40] Ruan X., Luo J., Zhang P., Howell K. (2022). The salivary microbiome shows a high prevalence of core bacterial members yet variability across human populations. npj Biofilms Microbiomes.

[41] Collado M.C., Engen P.A., Bandín C., Cabrera-Rubio R., Voigt R.M., Green S.J., Naqib A., Keshavarzian A., Scheer F.A.J.L., Garaulet M. (2018). Timing of food intake impacts daily rhythms of human salivary microbiota: A randomized, crossover study. FASEB J..

[42] Kashyap B., Kullaa A.M. (2024). Salivary Metabolites Produced by Oral Microbes in Oral Diseases. Metabolites.

[43] Basic A., Dahlén G. (2023). Microbial metabolites in the pathogenesis of periodontal diseases: A narrative review. Front. Oral Health.

[44] Seshadri R., Myers G.S., Tettelin H., Eisen J.A., Heidelberg J.F., Dodson R.J., Davidsen T.M., DeBoy R.T., Fouts D.E., Haft D.H. (2004). Comparison of the genome of the oral pathogen Treponema denticola with other spirochete genomes. Proc. Natl. Acad. Sci. USA.

[45] Watanabe T., Matsuura M., Seto K. (1988). Proteolytic activities of Mycoplasma salivarium. Microbiol. Immunol..

[46] Barile M.F., Schimke R.T., Riggs D.B. (1966). Presence of the arginine dihydrolase pathway in Mycoplasma. J. Bacteriol..

[47] Barbour A., Philip K. (2022). Metabolites of the oral microbiome: Important mediators of multikingdom interactions. FEMS Microbiol. Rev..

[48] Bostanghadiri N., Kouhzad M., Taki E., Elahi Z., Khoshbayan A., Navidifar T., Darban-Sarokhalil D. (2024). Oral microbiota and metabolites: Key players in oral health and disorder, and microbiota-based therapies. Front. Microbiol..

[49] Nani B.D., Lima P.O., Marcondes F.K., Groppo F.C., Rolim G.S., Moraes A.B.A., Cogo-Müller K. (2017). Changes in salivary microbiota increase volatile sulfur compounds production in healthy male subjects with academic-related chronic stress. PLoS ONE.

[50] Fernández-Reina A., Urdiales J.L., Sánchez-Jiménez F. (2018). What We Know and What We Need to Know about Aromatic and Cationic Biogenic Amines in the Gastrointestinal Tract. Foods.

[51] Persson S., Edlund M.B., Claesson R., Carlsson J. (1990). The formation of hydrogen sulfide and methyl mercaptan by oral bacteria. Oral Microbiol. Immunol..

[52] Vasquez A.M., Mouchlis V.D., Dennis E.A. (2018). Review of four major distinct types of human phospholipase A2. Adv. Biol. Regul..

[53] Shah D.S.H., Russell R.R.B. (2004). A novel glucan-binding protein with lipase activity from the oral pathogen Streptococcus mutans. Microbiology.

[54] Sato H., Taketomi Y., Murakami M. (2016). Metabolic regulation by secreted phospholipase A_2_. Inflamm. Regen..

[55] Reisenberg M., Singh P.K., Williams G., Doherty P. (2012). The diacylglycerol lipases: Structure, regulation and roles in and beyond endocannabinoid signalling. Philos. Trans. R. Soc. B.

[56] Fernandez-Gutierrez M.M., Imangaliyev S., Prodan A., Loos B.G., Keijser B.J.F., Kleerebezem M. (2020). A salivary metabolite signature that reflects gingival host-microbe interactions: Instability predicts gingivitis susceptibility. Sci. Rep..

[57] Liu T., Zhang L., Joo D., Sun S.-C. (2017). NF-κB signaling in inflammation. Signal Transduct. Target. Ther..

[58] Schwandner R., Dziarski R., Wesche H., Rothe M., Kirschning C.J. (1999). Peptidoglycan- and lipoteichoic acid-induced cell activation is mediated by Toll-like receptor 2. J. Biol. Chem..

[59] Bauer S., Kirschning C.J., Häcker H., Redecke V., Hausmann S., Akira S., Wagner H., Lipford G.B. (2001). Human TLR9 confers responsiveness to bacterial DNA via species-specific CpG motif recognition. Proc. Natl. Acad. Sci. USA.

[60] Do T.T., Nguyen V.T., Nguyen N.T.N., Duong K.T.T., Nguyen T.T.M., Le D.N.T., Nguyen T.H. (2024). A Review of a Breakdown in the Barrier: Tight Junction Dysfunction in Dental Diseases. Clin. Cosmet. Investig. Dent..

[61] Zhang W., Luo J., Dong X., Zhao S., Hao Y., Peng C., Shi H., Zhou Y., Shan L., Sun Q. (2019). Salivary Microbial Dysbiosis is Associated with Systemic Inflammatory Markers and Predicted Oral Metabolites in Non-Small Cell Lung Cancer Patients. J. Cancer.

[62] Devaraja K., Aggarwal S. (2025). Dysbiosis of Oral Microbiome: A Key Player in Oral Carcinogenesis? A Critical Review. Biomedicines.

[63] Boksa F.A., Leinbach L.I., Mody D.P., Ganesan S.M., Mays J.W. (2025). Oral microbiome alterations after cancer treatment: A scoping review and analysis. Med. Oncol..

[64] Klymiuk I., Bilgilier C., Mahnert A., Prokesch A., Heininger C., Brandl I., Sahbegovic H., Singer C., Fuereder T., Steininger C. (2022). Chemotherapy-associated oral microbiome changes in breast cancer patients. Front. Oncol..

[65] Laheij A.M.G.A., Raber-Durlacher J.E., Koppelmans R.G.A., Huysmans M.-C.D.N.J.M., Potting C., van Leeuwen S.J.M., Hazenberg M.D., Brennan M.T., von Bültzingslöwen I., Johansson J.-E. (2019). Microbial changes in relation to oral mucositis in autologous hematopoietic stem cell transplantation recipients. Sci. Rep..

[66] Zaura E., Brandt B.W., Teixeira de Mattos M.J., Buijs M.J., Caspers M.P., Rashid M.U., Weintraub A., Nord C.E., Savell A., Hu Y. (2015). Same Exposure but Two Radically Different Responses to Antibiotics: Resilience of the Salivary Microbiome versus Long-Term Microbial Shifts in Feces. mBio.

[67] Hosseini M.S., Sanaie S., Mahmoodpoor A., Jabbari Beyrami S., Jabbari Beyrami H., Fattahi S., Jahanshahlou F., Zarei M., Rahimi Mamaghani A., Kuchaki Rafsanjani M. (2024). Cancer treatment-related xerostomia: Basics, therapeutics, and future perspectives. Eur. J. Med. Res..

[68] PDQ Supportive and Palliative Care Editorial Board (2024). Oral Complications of Cancer Therapies (PDQ^®^): Patient Version. PDQ Cancer Information Summaries.

[69] Hong B.Y., Sobue T., Choquette L., Dupuy A.K., Thompson A., Burleson J.A., Salner A.L., Schauer P.K., Joshi P., Fox E. (2019). Chemotherapy-induced oral mucositis is associated with detrimental bacterial dysbiosis. Microbiome.

[70] Staruch M., Speth M.M., Neyer P., Riesterer O., Aebersold D.M., Stieb S. (2024). Radiation-associated changes in saliva composition of head and neck cancer patients: A systematic review. Radiother. Oncol..

[71] Ingrosso G., Saldi S., Marani S., Wong A.Y.W., Bertelli M., Aristei C., Zelante T. (2021). Breakdown of Symbiosis in Radiation-Induced Oral Mucositis. J. Fungi.

[72] Bruno J.S., Al-Qadami G.H., Laheij A.M.G.A., Bossi P., Fregnani E.R., Wardill H.R. (2023). From Pathogenesis to Intervention: The Importance of the Microbiome in Oral Mucositis. Int. J. Mol. Sci..

[73] Klimiuk A., Zalewska A., Knapp M., Skutnik-Radziszewska A., Maciejczyk M. (2022). Could inflammation contribute to salivary gland dysfunction in patients with chronic heart failure?. Front. Immunol..

[74] Vieira G.S., Kimura T.C., Scarini J.F., de Lima-Souza R.A., Lavareze L., Emerick C., Gonçalves M.T., Damas I.I., Figueiredo-Maciel T., Sales de Sá R. (2024). Hematopoietic colony-stimulating factors in head and neck cancers: Recent advances and therapeutic challenges. Cytokine.

[75] Aldahlawi S., Youssef A.R., Shahabuddin S. (2018). Evaluation of chemokine CXCL10 in human gingival crevicular fluid, saliva, and serum as periodontitis biomarker. J. Inflamm. Res..

[76] Silva T.A., Garlet G.P., Fukada S.Y., Silva J.S., Cunha F.Q. (2007). Chemokines in Oral Inflammatory Diseases: Apical Periodontitis and Periodontal Disease. J. Dent. Res..

[77] Aggor F.E.Y., Bertolini M., Coleman B.M., Taylor T.C., Ponde N.O., Gaffen S.L. (2024). Combinatorial actions of IL-22 and IL-17 drive optimal immunity to oral candidiasis through SPRRs. PLoS Pathog..

[78] Jiménez C., Carvajal D., Hernández M., Valenzuela F., Astorga J., Fernández A. (2021). Levels of the interleukins 17A, 22, and 23 and the S100 protein family in the gingival crevicular fluid of psoriatic patients with or without periodontitis. An. Bras. Dermatol..

[79] Hotamisligil G.S. (2006). Inflammation and metabolic disorders. Nature.

[80] Rysman E., Brusselmans K., Scheys K., Timmermans L., Derua R., Munck S., Van Veldhoven P.P., Waltregny D., Daniëls V.W., Machiels J. (2010). De novo lipogenesis protects cancer cells from free radicals and chemotherapeutics by promoting membrane lipid saturation. Cancer Res..

[81] Lan T., Chen L., Wei X. (2021). Inflammatory cytokines in cancer: Comprehensive understanding and clinical progress in gene therapy. Cells.

[82] Bunte K., Beikler T. (2019). Th17 Cells and the IL-23/IL-17 Axis in the Pathogenesis of Periodontitis and Immune-Mediated Inflammatory Diseases. Int. J. Mol. Sci..

[83] Abusleme L., Moutsopoulos N.M. (2017). IL-17: Overview and role in oral immunity and microbiome. Oral Dis..

[84] Chen C., Zhang Q., Yu W., Chang B., Le A.D. (2020). Oral Mucositis: An Update on Innate Immunity and New Interventional Targets. J. Dent. Res..

[85] Damgaard C., Massarenti L., Danielsen A.K., Graversen J.H., Holmstrup P., Nielsen C.H., Palarasah Y. (2022). Complement component 3 and its activation split-products in saliva associate with periodontitis. J. Periodontol..

[86] Bhalla S.P., Shaju A.M., Figueredo C.M.d.S., Miranda L.A. (2022). Increased Levels of C5a in Gingival Crevicular Fluid and Saliva of Patients with Periodontal Disease. Pathogens.

[87] Brandtzaeg P. (2013). Secretory immunity with special reference to the oral cavity. J. Oral Microbiol..

[88] Vila T., Rizk A.M., Sultan A.S., Jabra-Rizk M.A. (2019). The power of saliva: Antimicrobial and beyond. PLoS Pathog..

[89] Oppenheim F.G., Xu T., McMillian F.M., Levitz S.M., Diamond R.D., Offner G.D., Troxler R.F. (1988). Histatins, a novel family of histidine-rich proteins in human parotid secretion. Isolation, characterization, primary structure, and fungistatic effects on Candida albicans. J. Biol. Chem..

[90] Greer A., Zenobia C., Darveau R.P. (2013). Defensins and LL-37: A review of function in the gingival epithelium. Periodontology.

[91] Gorr S.U. (2012). Antimicrobial peptides in periodontal innate defense. Front. Oral Biol..

[92] Johnstone K.F., Wei Y., Bittner-Eddy P.D., Vreeman G.W., Stone I.A., Clayton J.B., Reilly C.S., Walbon T.B., Wright E.N., Hoops S.L. (2021). Calprotectin (S100A8/A9) Is an Innate Immune Effector in Experimental Periodontitis. Infect. Immun..

[93] Kim H.D., Karna S., Shin Y., Vu H., Cho H.J., Kim S. (2021). S100A8 and S100A9 in saliva, blood and gingival crevicular fluid for screening established periodontitis: A cross-sectional study. BMC Oral Health.

[94] Marcotte H., Lavoie M.C. (1998). Oral microbial ecology and the role of salivary immunoglobulin A. Microbiol. Mol. Biol. Rev..

[95] Gallo M., Ferrari E., Giovati L., Pertinhez T.A., Artesani L., Conti S., Ciociola T. (2024). The Variability of the Salivary Antimicrobial Peptide Profile: Impact of Lifestyle. Int. J. Mol. Sci..

[96] Di Spirito F., Di Palo M.P., De Benedetto G., Piedepalumbo F., Galdi M., Cannatà D., Cafà N., Contaldo M. (2025). Periodontal Microbial Profiles Across Periodontal Conditions in Pediatric Subjects: A Narrative Review. Microorganisms.

[97] Kaczor-Urbanowicz K.E., Martin Carreras-Presas C., Aro K., Tu M., Garcia-Godoy F., Wong D.T. (2017). Saliva diagnostics: Current views and directions. Exp. Biol. Med..

[98] Kumari S., Samara M., Ampadi Ramachandran R., Gosh S., George H., Wang R., Pesavento R.P., Mathew M.T. (2024). A review on saliva-based health diagnostics: Biomarker selection and future directions. Biomed. Mater. Devices.

[99] Humphrey S.P., Williamson R.T. (2001). A review of saliva: Normal composition, flow, and function. J. Prosthet. Dent..

[100] Simińska E., Koba M. (2016). Amino acid profiling as a method of discovering biomarkers for early diagnosis of cancer. Amino Acids.

[101] Han Y., Jia L., Zheng Y., Li W. (2018). Salivary exosomes: Emerging roles in systemic disease. Int. J. Biol. Sci..

[102] Bastías D., Maturana A., Marín C., Martínez R., Niklander S.E. (2024). Salivary biomarkers for oral cancer detection: An exploratory systematic review. Int. J. Mol. Sci..

[103] Sarkar A., Kuehl M.N., Alman A.C., Burkhardt B.R. (2021). Linking the oral microbiome and salivary cytokine abundance to circadian oscillations. Sci. Rep..

[104] Kashyap B., Debnath N., Hsu Y.-H., Hu H.-C., Chen H.-Y. (2024). Salivary metabolites produced by oral microbes in oral cancer and oral potentially malignant disorder. Metabolites.

[105] Cleaver L.M., Carda-Diéguez M., Moazzez R., Carpenter G.H. (2023). Novel bacterial proteolytic and metabolic activity associated with dental erosion-induced oral dysbiosis. Microbiome.

[106] Gonzalez Agurto M., Olivares N., Canedo-Marroquin G., Espinoza D., Tortora S.C. (2024). The intersection of the oral microbiome and salivary metabolites in head and neck cancer: From diagnosis to treatment. Cancers.

[107] Radaic A., Kapila Y.L. (2021). The oralome and its dysbiosis: New insights into oral microbiome-host interactions. Comput. Struct. Biotechnol. J..

[108] Li Y., Ou Y., Fan K., Liu G. (2024). Salivary diagnostics: Opportunities and challenges. Theranostics.

[109] Lee M.G., Ohana E., Park H.W., Yang D., Muallem S. (2012). Molecular mechanism of pancreatic and salivary gland fluid and HCO_3_^−^ secretion. Physiol. Rev..

[110] Zhao H., Xu X., Diaz J., Muallem S. (1995). Na^+^, K^+^, and H^+^/HCO_3_^−^ transport in submandibular salivary ducts: Membrane localization of transporters. J. Biol. Chem..

[111] Carpenter G.H. (2013). The secretion, components, and properties of saliva. Annu. Rev. Food Sci. Technol..

[112] Ogobuiro I., Tuma F. (2026). Physiology, Renal. StatPearls.

[113] Hall J.E., do Carmo J.M., da Silva A.A., Wang Z., Hall M.E. (2019). Obesity, kidney dysfunction and hypertension: Mechanistic links. Nat. Rev. Nephrol..

[114] Ferrannini E. (2017). Sodium-glucose co-transporters and their inhibition: Clinical physiology. Cell Metab..

[115] Scott J.H., Menouar M.A., Dunn R.J. (2026). Physiology, Aldosterone. StatPearls.

[116] Proctor G.B. (2016). The physiology of salivary secretion. Periodontology.

[117] Kubala E., Strzelecka P., Grzegocka M., Lietz-Kijak D., Gronwald H., Skomro P., Kijak E. (2018). A review of selected studies that determine the physical and chemical properties of saliva in the field of dental treatment. BioMed Res. Int..

[118] Imenez Silva P.H., Mohebbi N. (2022). Kidney metabolism and acid-base control: Back to the basics. Pflüg. Arch. Eur. J. Physiol..

[119] Hopkins E., Sanvictores T., Sharma S. (2026). Physiology, Acid Base Balance. StatPearls.

[120] Bardow A., Moe D., Nyvad B., Nauntofte B. (2000). The buffer capacity and buffer systems of human whole saliva measured without loss of CO_2_. Arch. Oral Biol..

[121] Hogan P.G., Lewis R.S., Rao A. (2010). Molecular basis of calcium signaling in lymphocytes: STIM and ORAI. Annu. Rev. Immunol..

[122] Vig M., Kinet J.P. (2009). Calcium signaling in immune cells. Nat. Immunol..

[123] Romani A.M.P., Vink R., Nechifor M. (2011). Intracellular Magnesium Homeostasis. Magnesium in the Central Nervous System.

[124] Singhal I., Arora M., Dave A., Bansal S.K., Saluja P., Rai R. (2023). Evaluation of magnesium levels in serum and saliva by calmagite method in individuals with tobacco habits with or without potentially malignant disorders. J. Oral Maxillofac. Pathol..

[125] Lima F.D.S., Fock R.A. (2020). A review of the action of magnesium on several processes involved in the modulation of hematopoiesis. Int. J. Mol. Sci..

[126] Dongiovanni P., Meroni M., Casati S., Goldoni R., Thomaz D.V., Kehr N.S., Galimberti D., Del Fabbro M., Tartaglia G.M. (2023). Salivary biomarkers: Novel noninvasive tools to diagnose chronic inflammation. Int. J. Oral Sci..

[127] Berardi R., Torniai M., Lenci E., Pecci F., Morgese F., Rinaldi S. (2019). Electrolyte disorders in cancer patients: A systematic review. J. Cancer Metastasis Treat..

[128] Munsif A., Nowaczyk A., Fijałkowski Ł., Riaz S., Jamil A. (2025). Salivary biomarkers in cancer detection and management. Acta Biochim. Pol..

[129] Bennet D., Khorsandian Y., Pelusi J., Mirabella A., Pirrotte P., Zenhausern F. (2021). Molecular and physical technologies for monitoring fluid and electrolyte imbalance: A focus on cancer population. Clin. Transl. Med..

[130] Dong Y., Wang T., Wu H. (2024). The role of cytokines from salivary gland epithelial cells in the immunopathology of Sjögren’s syndrome. Front. Immunol..

[131] D’Agostino C., Elkashty O.A., Chivasso C., Perret J., Tran S.D., Delporte C. (2020). Insight into salivary gland aquaporins. Cells.

[132] Baker O.J., Camden J.M., Redman R.S., Jones J.E., Seye C.I., Erb L., Weisman G.A. (2008). Proinflammatory cytokines tumor necrosis factor-alpha and interferon-gamma alter tight junction structure and function in the rat parotid gland Par-C10 cell line. Am. J. Physiol.-Cell Physiol..

[133] Owecki W., Wojtowicz K., Nijakowski K. (2025). Salivary extracellular vesicles in detection of cancers other than head and neck: A systematic review. Cells.

[134] Shetty S.R., Babu S., Kumari S., Shetty P., Hegde S., Karikal A. (2015). Status of trace elements in saliva of oral precancer and oral cancer patients. J. Cancer Res. Ther..

[135] Saavedra J.A., Novo D.R., Mesko M.F., Uchoa Vasconcellos A.C., Duarte Da Silva K., Rojas Zuñiga G., Ramires R.F., Tarquinio S.B.C. (2022). Comparison of salivary electrolytes profile in oral potentially malignant disorders and oral squamous cell carcinoma. Asian Pac. J. Cancer Prev..

[136] Nijakowski K., Zdrojewski J., Nowak M., Gruszczyński D., Knoll F., Surdacka A. (2022). Salivary metabolomics for systemic cancer diagnosis: A systematic review. Metabolites.

[137] Aps J.K.M., Martens L.C. (2005). Review: The physiology of saliva and transfer of drugs into saliva. Forensic Sci. Int..

[138] Al Habobe H., Haverkort E.B., Nazmi K., Van Splunter A.P., Pieters R.H.H., Bikker F.J. (2024). The impact of saliva collection methods on measured salivary biomarker levels. Clin. Chim. Acta.

[139] Wei Z., Liu X., Cheng C., Yu W., Yi P. (2021). Metabolism of amino acids in cancer. Front. Cell Dev. Biol..

[140] Sirniö P., Väyrynen J.P., Klintrup K., Mäkelä J., Karhu T., Herzig K.H., Minkkinen I., Mäkinen M.J., Karttunen T.J., Tuomisto A. (2019). Alterations in serum amino-acid profile in the progression of colorectal cancer: Associations with systemic inflammation, tumour stage and patient survival. Br. J. Cancer.

[141] Martindale R.G., Heyland D.K., Rugeles S.J., Wernerman J., Weijs P.J., Patel J.J., McClave S.A. (2017). Protein kinetics and metabolic effects related to disease states in the intensive care unit. Nutr. Clin. Pract..

[142] Durham W.J., Dillon E.L., Sheffield-Moore M. (2009). Inflammatory burden and amino acid metabolism in cancer cachexia. Curr. Opin. Clin. Nutr. Metab. Care.

[143] Lieu E.L., Nguyen T., Rhyne S., Kim J. (2020). Amino acids in cancer. Exp. Mol. Med..

[144] Dyachenko E.I., Bel’skaya L.V. (2024). Transmembrane amino acid transporters in shaping the metabolic profile of breast cancer cell lines: The focus on molecular biological subtype. Curr. Issues Mol. Biol..

[145] Chen C.-L., Hsu S.-C., Chung T.-Y., Chu C.-Y., Wang H.-J., Hsiao P.-W. (2021). Arginine signaling and cancer metabolism. Cancers.

[146] Gallo O., Fini-Storchi I., Vergari W.A., Masini E., Morbidelli L., Ziche M., Franchi A. (1998). Role of nitric oxide in angiogenesis and tumor progression in head and neck cancer. JNCI J. Natl. Cancer Inst..

[147] Lian G., Gnanaprakasam J.R., Wang T., Wu R., Chen X., Liu L., Shen Y., Yang M., Yang J., Chen Y. (2018). Glutathione de novo synthesis but not recycling process coordinates with glutamine catabolism to control redox homeostasis and directs murine T cell differentiation. eLife.

[148] Hasan T., Arora R., Bansal A.K., Bhattacharya R., Sharma G.S., Singh L.R. (2019). Disturbed homocysteine metabolism is associated with cancer. Exp. Mol. Med..

[149] Akbari Z., Dijojin R.T., Zamani Z., Hosseini R.H., Arjmand M. (2021). Aromatic amino acids play a harmonizing role in prostate cancer: A metabolomics-based cross-sectional study. Int. J. Reprod. Biomed..

[150] Opitz C.A., Somarribas Patterson L.F., Mohapatra S.R., Dewi D.L., Sadik A., Platten M., Trump S. (2020). The therapeutic potential of targeting tryptophan catabolism in cancer. Br. J. Cancer.

[151] Helmerhorst E.J., Dawes C., Oppenheim F.G. (2018). The complexity of oral physiology and its impact on salivary diagnostics. Oral Dis..

[152] Hyvärinen E., Kashyap B., Kullaa A.M. (2023). Oral sources of salivary metabolites. Metabolites.

[153] Yoshizawa J.M., Schafer C.A., Schafer J.J., Farrell J.J., Paster B.J., Wong D.T. (2013). Salivary biomarkers: Toward future clinical and diagnostic utilities. Clin. Microbiol. Rev..

[154] Syrjänen S.M., Alakuijala L., Alakuijala P., Markkanen S.O., Markkanen H. (1990). Free amino acid levels in oral fluids of normal subjects and patients with periodontal disease. Arch. Oral Biol..

[155] Subbarao K.C., Nattuthurai G.S., Sundararajan S.K., Sujith I., Joseph J., Syedshah Y.P. (2019). Gingival crevicular fluid: An overview. J. Pharm. BioAllied Sci..

[156] Balci N., Kurgan Ş., Çekici A., Çakır T., Serdar M.A. (2021). Free amino acid composition of saliva in patients with healthy periodontium and periodontitis. Clin. Oral Investig..

[157] Hasan M.M., Razu M.H., Akter S., Mou S.A., Islam M., Khan M. (2024). Development and validation of a non-invasive method for quantifying amino acids in human saliva. RSC Adv..

[158] Di Pietro L., Boroumand M., Lattanzi W., Manconi B., Salvati M., Cabras T., Olianas A., Flore L., Serrao S., Calò C.M. (2023). A catalog of coding sequence variations in salivary proteins’ genes occurring during recent human evolution. Int. J. Mol. Sci..

[159] Schwerdt G., Schulz M.C., Kopf M., Mildenberger S., Reime S., Gekle M. (2025). Physiological regulation of oral saliva ion composition and flow rate are not coupled in healthy humans—Partial revision of our current knowledge required. Pflüg. Arch. Eur. J. Physiol..

[160] Kaufman E., Lamster I.B. (2002). The diagnostic applications of saliva—A review. Crit. Rev. Oral Biol. Med..

[161] Ferrari E., Gallo M., Spisni A., Antonelli R., Meleti M., Pertinhez T.A. (2023). Human serum and salivary metabolomes: Diversity and closeness. Int. J. Mol. Sci..

[162] Haeckel R., Hanecke P. (1996). The application of saliva, sweat and tear fluid for diagnostic purposes. Ann. Biol. Clin..

[163] Messana I., Manconi B., Cabras T., Boroumand M., Sanna M.T., Iavarone F., Olianas A., Desiderio C., Rossetti D.V., Vincenzoni F. (2023). The post-translational modifications of human salivary peptides and proteins evidenced by top-down platforms. Int. J. Mol. Sci..

[164] Vitorino R., Alves R., Barros A., Caseiro A., Ferreira R., Lobo M.C., Bastos A., Duarte J., Carvalho D., Santos L.L. (2010). Finding new posttranslational modifications in salivary proline-rich proteins. Proteomics.

[165] Ruhl S. (2012). The scientific exploration of saliva in the post-proteomic era: From database back to basic function. Expert Rev. Proteom..

[166] Nijakowski K., Gruszczyński D., Kopała D., Surdacka A. (2022). Salivary metabolomics for systemic cancer diagnosis. Int. J. Mol. Sci..

[167] Wang X., Kaczor-Urbanowicz K.E., Wong D.T.W. (2017). Salivary biomarkers in cancer detection. Med. Oncol..

[168] Lai H.S., Lee J.C., Lee P.H., Wang S.T., Chen W.J. (2005). Plasma free amino acid profile in cancer patients. Semin. Cancer Biol..

[169] Lau C., Kim Y., Chia D., Spielmann N., Eibl G., Elashoff D., Wei F., Lin Y.L., Moro A., Grogan T. (2013). Role of pancreatic cancer-derived exosomes in salivary biomarker development. J. Biol. Chem..

[170] Nonaka T., Wong D.T.W. (2017). Saliva-exosomics in cancer: Molecular characterization of cancer-derived exosomes in saliva. Enzymes.

[171] Setiawan T., Sari I.N., Wijaya Y.T., Julianto N.M., Muhammad J.A., Lee H., Chae J.H., Kwon H.Y. (2023). Cancer cachexia: Molecular mechanisms and treatment strategies. J. Hematol. Oncol..

[172] Woo J.S., Hwang S.H., Yang S.C., Lee K.H., Lee Y.S., Choi J.W., Park J.S., Jhun J.Y., Park S.H., Cho M.L. (2023). Lactobacillus acidophilus and propionate attenuate Sjögren’s syndrome by modulating the STIM1-STING signaling pathway. Cell Commun. Signal..

[173] Kim D.S., Woo J.S., Min H.K., Choi J.W., Moon J.H., Park M.J., Kwok S.K., Park S.H., Cho M.L. (2021). Short-chain fatty acid butyrate induces IL-10-producing B cells by regulating circadian-clock-related genes to ameliorate Sjögren’s syndrome. J. Autoimmun..

[174] Cong X., Mao X.D., Wu L.L., Yu G.Y. (2024). The role and mechanism of tight junctions in the regulation of salivary gland secretion. Oral Dis..

[175] Solomatin D.V., Sarf E.A., Bel’skaya L.V. (2025). Metabolic Features of Saliva Before and After Breast Cancer Surgery. Metabolites.

[176] Ishikawa S., Sugimoto M., Konta T., Kitabatake K., Ueda S., Edamatsu K., Okuyama N., Yusa K., Iino M. (2022). Salivary Metabolomics for Prognosis of Oral Squamous Cell Carcinoma. Front. Oncol..

[177] Neyraud E., Cabaret S., Brignot H., Chabanet C., Labouré H., Guichard E., Berdeaux O. (2017). The basal free fatty acid concentration in human saliva is related to salivary lipolytic activity. Sci. Rep..

[178] Wu J., Liu G., Jia R., Guo J. (2023). Salivary extracellular vesicles: Biomarkers and beyond in human diseases. Int. J. Mol. Sci..

[179] Cui L., Zheng J., Lu Y., Lin P., Lin Y., Zheng Y., Xu R., Mai Z., Guo B., Zhao X. (2024). New frontiers in salivary extracellular vesicles: Transforming diagnostics, monitoring, and therapeutics in oral and systemic diseases. J. Nanobiotechnol..

[180] Caterino M., Fedele R., Carnovale V., Castaldo A., Gelzo M., Iacotucci P., Ruoppolo M., Castaldo G. (2023). Lipidomic alterations in human saliva from cystic fibrosis patients. Sci. Rep..

[181] Agatonovic-Kustrin S., Morton D.W., Smirnov V., Petukhov A., Gegechkori V., Kuzina V., Gorpinchenko N., Ramenskaya G. (2019). Analytical strategies in lipidomics for discovery of functional biomarkers from human saliva. Dis. Markers.

[182] Matczuk J., Żendzian-Piotrowska M., Maciejczyk M., Kurek K. (2017). Salivary lipids: A review. Adv. Clin. Exp. Med..

[183] Verstappen G.M., Pringle S., Bootsma H., Kroese F.G.M. (2021). Epithelial-immune cell interplay in primary Sjögren syndrome salivary gland pathogenesis. Nat. Rev. Rheumatol..

[184] Liao R., Yang H.T., Li H., Liu L.X., Li K., Li J.J., Liang J., Hong X.P., Chen Y.L., Liu D.Z. (2022). Recent advances of salivary gland biopsy in Sjögren’s syndrome. Front. Med..

[185] Fineide F., Chen X., Bjellaas T., Vitelli V., Utheim T.P., Jensen J.L., Galtung H.K. (2021). Characterization of lipids in saliva, tears and minor salivary glands of Sjögren’s syndrome patients using an HPLC/MS-based approach. Int. J. Mol. Sci..

[186] Gao K., Zhou H., Zhang L., Lee J.W., Zhou Q., Hu S., Wolinsky L.E., Farrell J., Eibl G., Wong D.T.W. (2009). Systemic disease-induced salivary biomarker profiles in mouse models of melanoma and non-small cell lung cancer. PLoS ONE.

[187] Klein A., Klein J., Chacham M., Kleinman S., Shuster A., Peleg O., Ianculovici C., Kaplan I. (2022). Acinar atrophy, fibrosis and fatty changes are significantly more common than Sjogren’s syndrome in minor salivary gland biopsies. Medicina.

[188] Skarstein K., Aqrawi L.A., Øijordsbakken G., Jonsson R., Jensen J.L. (2016). Adipose tissue is prominent in salivary glands of Sjögren’s syndrome patients and appears to influence the microenvironment in these organs. Autoimmunity.

[189] Yin H., Pranzatelli T.J.F., French B.N., Zhang N., Warner B.M., Chiorini J.A. (2021). Sclerosing sialadenitis is associated with salivary gland hypofunction and a unique gene expression profile in Sjögren’s syndrome. Front. Immunol..

[190] Shikama Y., Ishimaru N., Kudo Y., Bando Y., Aki N., Hayashi Y., Funaki M. (2013). Effects of free fatty acids on human salivary gland epithelial cells. J. Dent. Res..

[191] Mena S.J., Manosalva C., Carretta M.D., Teuber S., Olmo I., Burgos R.A., Hidalgo M.A. (2016). Differential free fatty acid receptor-1 (FFAR1/GPR40) signalling is associated with gene expression or gelatinase granule release in bovine neutrophils. Innate Immun..

[192] Hidalgo M.A., Carretta M.D., Burgos R.A. (2021). Long chain fatty acids as modulators of immune cells function: Contribution of FFA1 and FFA4 receptors. Front. Physiol..

[193] Korbecki J., Bajdak-Rusinek K. (2019). The effect of palmitic acid on inflammatory response in macrophages: An overview of molecular mechanisms. Inflamm. Res..

[194] Belstrøm D., Damgaard C., Könönen E., Gürsoy M., Holmstrup P., Gürsoy U.K. (2017). Salivary cytokine levels in early gingival inflammation. J. Oral Microbiol..

[195] Diesch T., Filippi C., Fritschi N., Filippi A., Ritz N. (2021). Cytokines in saliva as biomarkers of oral and systemic oncological or infectious diseases: A systematic review. Cytokine.

[196] Ewert P., Aguilera S., Alliende C., Kwon Y.J., Albornoz A., Molina C., Urzúa U., Quest A.F.G., Olea N., Pérez P. (2010). Disruption of tight junction structure in salivary glands from Sjögren’s syndrome patients is linked to proinflammatory cytokine exposure. Arthritis Rheum..

[197] Mao X., Li H., Min S., Su J., Wei P., Zhang Y., He Q., Wu L., Yu G., Cong X. (2025). Loss of tricellular tight junction tricellulin leads to hyposalivation in Sjögren’s syndrome. Int. J. Oral Sci..

[198] Fung K.Y.Y., Fairn G.D., Lee W.L. (2018). Transcellular vesicular transport in epithelial and endothelial cells: Challenges and opportunities. Traffic.

[199] Qi W., Tian J., Wang G., Yan Y., Wang T., Wei Y., Wang Z., Zhang G., Zhang Y., Wang J. (2024). Advances in cellular and molecular pathways of salivary gland damage in Sjögren’s syndrome. Front. Immunol..

[200] Zhao T., Zhang R., Li Z., Qin D., Wang X. (2024). A comprehensive review of Sjögren’s syndrome: Classification criteria, risk factors, and signaling pathways. Heliyon.

[201] Porcheri C., Mitsiadis T.A. (2019). Physiology, Pathology and Regeneration of Salivary Glands. Cells.

[202] Chibly A.M., Aure M.H., Patel V.N., Hoffman M.P. (2022). Salivary gland function, development, and regeneration. Physiol. Rev..

[203] Bibi T., Khurshid Z., Rehman A., Imran E., Srivastava K.C., Shrivastava D. (2021). Gingival Crevicular Fluid (GCF): A Diagnostic Tool for the Detection of Periodontal Health and Diseases. Molecules.

[204] Yue Y., Liu Q., Xu C., Loo W.T., Wang M., Wen G., Cheung M.N., Bai L.J., Dou Y.D., Chow L.W. (2013). Comparative evaluation of cytokines in gingival crevicular fluid and saliva of patients with aggressive periodontitis. Int. J. Biol. Markers.

[205] Liu Y., Zhao R., Reda B., Yang W., Hannig M., Qu B. (2021). Profiling of cytokines, chemokines and growth factors in saliva and gingival crevicular fluid. Cytokine.

[206] Paganini A., Fritschi N., Filippi C., Ritz N., Simmen U., Scheinemann K., Filippi A., Diesch-Furlanetto T. (2025). Comparative analysis of salivary cytokine profiles in newly diagnosed pediatric patients with cancer and healthy children. Sci. Rep..

[207] Chauhan A., Yadav S.S., Dwivedi P., Lal N., Usman K., Khattri S. (2016). Correlation of Serum and Salivary Cytokines Level with Clinical Parameters in Metabolic Syndrome with Periodontitis. J. Clin. Lab. Anal..

[208] Parkin G.M., Kim S., Mikhail A., Malhas R., McMillan L., Hollearn M., Granger D.A., Mapstone M., Yassa M.A., Thomas E.A. (2023). Associations between saliva and plasma cytokines in cognitively normal, older adults. Aging Clin. Exp. Res..

[209] Metgud R., Bajaj S. (2016). Altered serum and salivary C-reactive protein levels in patients with oral premalignant lesions and oral squamous cell carcinoma. Biotech. Histochem..

[210] Shan J., Sun Z., Yang J., Xu J., Shi W., Wu Y., Fan Y., Li H. (2019). Discovery and preclinical validation of proteomic biomarkers in saliva for early detection of oral squamous cell carcinomas. Oral Dis..

[211] Deepthi G., Nandan S.R.K., Kulkarni P.G. (2019). Salivary tumour necrosis factor-α as a biomarker in oral leukoplakia and oral squamous cell carcinoma. Asian Pac. J. Cancer Prev..

[212] Li M., Li M., Qiao L., Wu C., Xu D., Zhao Y., Zeng X. (2023). Role of JAK-STAT signaling pathway in pathogenesis and treatment of primary Sjögren’s syndrome. Chin. Med. J..

[213] Spielmann N., Wong D.T. (2011). Saliva: Diagnostics and therapeutic perspectives. Oral Dis..

[214] Cristaldi M., Mauceri R., Di Fede O., Giuliana G., Campisi G., Panzarella V. (2019). Salivary Biomarkers for Oral Squamous Cell Carcinoma Diagnosis and Follow-Up: Current Status and Perspectives. Front. Physiol..

[215] Matsuoka M., Soria S.A., Pires J.R., Sant’Ana A.C.P., Freire M. (2025). Natural and induced immune responses in oral cavity and saliva. BMC Immunol..

[216] Chiabotto G., Gai C., Deregibus M.C., Camussi G. (2019). Salivary Extracellular Vesicle-Associated exRNA as Cancer Biomarker. Cancers.

[217] Derakhshan S., Poosti A., Razavi A.E., Moosavi M.A., Mahdavi N., Naieni F.B., Hesari K.K., Rahpeima A. (2021). Evaluation of squamous cell carcinoma antigen 1 expression in oral squamous cell carcinoma (tumor cells and peritumoral T-lymphocytes) and verrucous carcinoma and comparison with normal oral mucosa. J. Appl. Oral Sci..

[218] Rajguru J.P., Mouneshkumar C.D., Radhakrishnan I.C., Negi B.S., Maya D., Hajibabaei S., Rana V. (2020). Tumor markers in oral cancer: A review. J. Fam. Med. Prim. Care.

[219] Geng X.F., Du M., Han J.X., Zhang M., Tang X.F., Xing R.D. (2013). Saliva CA125 and TPS levels in patients with oral squamous cell carcinoma. Int. J. Biol. Markers.

[220] Nagler R., Bahar G., Shpitzer T., Feinmesser R. (2006). Concomitant analysis of salivary tumor markers--a new diagnostic tool for oral cancer. Clin. Cancer Res..

[221] Rancourt C., Matte I., Lane D., Piché A. (2012). The Role of MUC16 Mucin (CA125) in the Pathogenesis of Ovarian Cancer. Ovarian Cancer—Basic Science Perspective.

[222] Kim S., Park B.K., Seo J.H., Choi J., Choi J.W., Lee C.K., Chung J.B., Park Y., Kim D.W. (2020). Carbohydrate antigen 19-9 elevation without evidence of malignant or pancreatobiliary diseases. Sci. Rep..

[223] Lee T., Teng T.Z.J., Shelat V.G. (2020). Carbohydrate antigen 19-9—Tumor marker: Past, present, and future. World J. Gastrointest. Surg..

[224] Galgano M., Hampton G., Frierson H. (2006). Comprehensive analysis of HE4 expression in normal and malignant human tissues. Mod. Pathol..

[225] Bingle L., Cross S.S., High A.S., Wallace W.A., Rassl D., Yuan G., Hellstrom I., Campos M.A., Bingle C.D. (2006). WFDC2 (HE4): A potential role in the innate immunity of the oral cavity and respiratory tract and the development of adenocarcinomas of the lung. Respir. Res..

[226] Wolber P., Mayer M., Nachtsheim L., Prinz J., Klußmann J.P., Quaas A., Arolt C. (2022). Expression of Mucins in Different Entities of Salivary Gland Cancer: Highest Expression of Mucin-1 in Salivary Duct Carcinoma: Mucin-1—Highest expression in Salivary Duct Carcinoma. Head Neck Pathol..

[227] Khurshid Z., Zafar M.S., Khan R.S., Najeeb S., Slowey P.D., Rehman I.U. (2018). Role of Salivary Biomarkers in Oral Cancer Detection. Adv. Clin. Chem..

[228] Uhlén M., Fagerberg L., Hallström B.M., Lindskog C., Oksvold P., Mardinoglu A., Sivertsson Å., Kampf C., Sjöstedt E., Asplund A. (2015). Tissue-based map of the human proteome. Science.

[229] Human Protein Atlas. https://www.proteinatlas.org.

[230] Hernández M., Baeza M., Contreras J., Sorsa T., Tervahartiala T., Valdés M., Chaparro A., Hernández-Ríos P. (2020). MMP-8, TRAP-5, and OPG Levels in GCF Diagnostic Potential to Discriminate between Healthy Patients’, Mild and Severe Periodontitis Sites. Biomolecules.

[231] Tatullo M., Codispoti B., Makeeva I., Benincasa C., Spagnuolo G. (2019). From Mouth to Brain: Neuroendocrine Markers Play as a Crosstalk Among Oral and Neurodegenerative Diseases. Front. Endocrinol..

[232] Cobo T., Obaya A., Cal S., Solares L., Cabo R., Vega J.A., Cobo J. (2015). Immunohistochemical localization of periostin in human gingiva. Eur. J. Histochem..

[233] Liu Y., Wei C., Wang S., Ding S., Li Y., Li Y., Zhang D., Zhu G., Meng Z. (2023). Role of prognostic gene DKK1 in oral squamous cell carcinoma. Oncol. Lett..

[234] Kawai R., Sugita Y., Suzumura T., Hattori T., Yoshida W., Kubo K., Maeda H. (2021). Melanoma Inhibitory Activity and Melanoma Inhibitory Activity 2 as Novel Immunohistochemical Markers of Oral Epithelial Dysplasia. J. Clin. Med..

[235] Al-Chalabi M., Bass A.N., Alsalman I. (2026). Physiology, Prolactin. StatPearls.

